# Cyanine Nanocages Activated by Near-Infrared Light for the Targeted Treatment of Traumatic Brain Injury

**DOI:** 10.3389/fchem.2020.00769

**Published:** 2020-08-31

**Authors:** Caroline E. Black, Eugene Zhou, Caitlin M. DeAngelo, Isaac Asante, Stan G. Louie, Nicos A. Petasis, Mark S. Humayun

**Affiliations:** ^1^Department of Chemistry, University of Southern California, Los Angeles, CA, United States; ^2^School of Pharmacy, University of Southern California, Los Angeles, CA, United States; ^3^Ginsburg Institute for Biomedical Therapeutics, University of Southern California, Los Angeles, CA, United States; ^4^Keck School of Medicine, Viterbi School of Engineering, and Roski Eye Institute, University of Southern California, Los Angeles, CA, United States

**Keywords:** cyanine dye, near-infrared, light-activated, traumatic brain injury (TBI), gabapentin

## Abstract

Traumatic brain injury (TBI) is a common and prevalent condition that affects large numbers of people across a range of ages. Individuals engaging in physical activities and victims of accidents are at a higher risk for TBI. There is a lack of available treatment specifically for TBI. Given the difficulty to determine its precise location in the brain, TBI remains difficult to fully diagnose or treat. Herein, we disclose a novel strategy for directing therapeutic agents to TBI sites, without the need to determine the precise location of the TBI activity in the brain. This novel approach is based on the use of a cyanine dye nanocage carrying Gabapentin, a known TBI therapeutic agent. Upon exposure of the cyanine nanocage to near-infrared light, the local release of Gabapentin is triggered, selectively at the TBI-affected site.

## Introduction

Each year, more people suffer from traumatic brain injury (TBI) than breast cancer, HIV/AIDS infections, multiple sclerosis, and spinal cord injuries combined (Naeser et al., 2016). The economic burden to treat TBI and the long-term sequalae have been estimated to be $60–76.5 billion annually (Naeser et al., 2016). There are a variety of medical interventions used to treat TBI, including cranial elevation, hyperosmolar therapy, therapeutic hypothermia, and surgical decompression (Galgano et al., 2017).

Unfortunately, about one-third of patients do not survive because of complications from secondary injury. The initial TBI can activate secondary mechanisms leading to additional injury(ies) responsible for continual neuronal damage. Secondary injury in TBI is a consequence of inflammatory and neuroexcitatory processes that cause further damage after the primary physical damage.

This cascade can include ionic disturbance (i.e., elevated intracellular Ca^2+^ levels), excitotoxicity (elevated extracellular glutamate levels), mitochondrial dysfunction, oxidative stress, neuroinflammation, blood-brain-barrier (BBB) damage, and cell death (Tran, 2014; Pearn et al., 2017). Secondary injuries can begin minutes to days after the primary injury and can last for years following the accident. If unresolved, these secondary injuries can cause long-term effects such as cognitive and attention deficits, poor sensory processing and communication, depression, and anxiety (Tran, 2014; Galgano et al., 2017; Pearn et al., 2017). Due to the significant long-term brain damage beginning just minutes after the primary injury, treatment for TBI needs to promote a rapid response to prevent the neuroinflammatory cascade required to promote secondary injury.

Currently there is no approved treatment for TBI. Even with the lack of FDA-approved therapies targeting these mechanisms, there are several therapeutic options to treat secondary injury that were shown to be effective in animal models. This includes calcium channel blockers (e.g., Gabapentin) and NMDA receptor antagonists (Dexanabinol), glutamate receptor antagonists (Topiramate, Phencyclidine, Dextromethorphan), free-radical scavengers (Tirilazad, dropped during Phase III), the neuroprotective glycoprotein erythropoietin, the calcium-binding protein S100B, stem cell therapy, and cyclosporin-A (Pearn et al., 2017).

**Figure 1:**
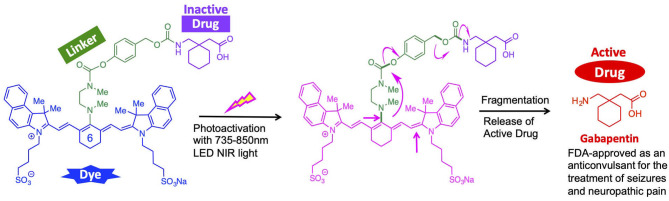
NIR light-activated drug delivery mechanism for the treatment of TBI.

Gabapentin (Figure 1) is a γ-aminobutyric acid (GABA) analog that is FDA approved as an anticonvulsant for the treatment of seizures, neuropathic pain, and other neurologic conditions (Kukkar et al., 2013). It also has the potential to correct the elevated levels of intracellular calcium exhibited in cases of TBI. Gabapentin has no activity on GABAA or GABAB receptors with regards to GABA uptake.

However, it has high-affinity binding onto gabapentin binding protein (GBP). Gabapentin binds onto the α_2_δ subunit of an L-type voltage-dependent Ca^2+^ channel (Offord and Isom, 2016) which is an auxiliary subunit of voltage-sensitive Ca^2+^ channels.

One effective approach to treating TBI is to ameliorate the initiation of inflammatory responses. This strategy requires the administration within the first or “golden” hour after the initial injury to prevent the onset of late clinical sequalae (Pearn et al., 2017). This proposed system must be stable, easily transportable and simple to use by first responders. Moreover, this integrative system must be highly specific and able to localize at the injured site(s), thus circumventing need for *a priori* CT or MRI imaging to identify the location and extent of the injury.

Thus, there is a dire need for a safe and effective treatment of TBI administered within the “golden hour” after the primary injury. In an effort to fill this void, herein we report the design and synthesis of a cyanine-based nanocage system that carries caged Gabapentin, which is designed to selectively release at the injured brain site upon exposure to Near-Infrared (NIR) light. The use of a light-activated nanocage for the delivery of a therapeutic provides two major benefits: targeted delivery of therapy at injured site and improved therapeutic window of future drug cargo. Notably, our approach does not require that we first identify the precise brain location of the TBI sites. Indocyanine-based nanocages were chosen due to their optical characteristics (λ_max_ ~830 nm in blood) and safety features (Luo et al., 2011; Mohammad et al., 2013). These physicochemical features will be sensitive to NIR light-activated release to selectively release Gabapentin at the TBI sites.

The concept of a photoactivated nanocage has wide applications in diverse chemical areas, such as drug delivery, cell imaging, antibody drug conjugates, and materials science (Ma et al., 2008; Gorka et al., 2015, 2018a,b; Owens et al., 2015; Wu et al., 2015; Gorka and Schnermann, 2016). A substantial challenge in this field, however, has been the requirement for the use of a high energy light source, e.g., UV or blue light, which is associated with high toxicity, background absorbance in biological media, and limited tissue penetration (Gorka et al., 2014).

In recent years, a number of publications have explored the use of NIR light (690 nm) to initiate the release of caged drug candidates upon photochemical activation (Gorka et al., 2014, 2018a; Nani et al., 2015c; Patel et al., 2016; Yamamoto et al., 2019). This process triggers the fragmentation of the connecting linker groups, thereby prompting the release of the active drug. A similar drug release approach was developed for molecular systems that include a photoactivatable NIR dye (780 nm), connected via a linker to a caged toxic drug (Duocarmycin), and a second linker connecting to an antibody (Nani et al., 2015a, 2017; Sato et al., 2015, 2016). The use of antibody-drug conjugates has the advantage of protecting the toxic drug cargo, until activation by NIR light and release at the photoactivated site.

Heptamethine cyanine dyes were traditionally used as reporter molecules that bind to biomolecules such as proteins, RNA, and DNA (Lipowska et al., 1993; Williams et al., 1993, 1997; Shealy et al., 1995; Christian Mason et al., 1997; Sowell et al., 2001a,b; Strekowski et al., 2001; Patonay et al., 2004; Luciano et al., 2019). More recently, the use of a cyanine dye as a photoactivated nanocage for targeted drug release has been reported by Gorka et al. (2014), Nani et al. (2015a,c, 2017), Sato et al. (2015, 2016), Patel et al. (2016) and Yamamoto et al. (2019). Cyanine dyes offer several advantages such as maximum absorbances in the NIR window (650–900 nm), low background absorbance interference, and low toxicity (Marshall et al., 2010; Donald et al., 2011). These features complement NIR light sources with excellent tissue and bone penetration, with an increased safety profile when compared to lower wavelength light (Naeser et al., 2016). Specifically, NIR light (850 nm) penetrates across bone, extracellular fluid, and tissue at a depth of >6 cm (Yue and Humayun, 2015).

Herein, we report an indocyanine-based NIR light-activated drug delivery strategy for the treatment of TBI (Figure 1). The dye scaffold is based on indocyanine green (ICG), an FDA-approved dye with a good safety profile, aqueous solubility, and maximum absorption of low-energy light close to 830 nm. Our synthetic approach is based on a three-component cyanine-based nanocage, consisting of the cyanine dye (blue), that is chemically linked to a fragmentable diamine linker unit (green), connected to the caged (inactive) TBI drug (purple).

Our design utilizes cyanine dyes with maximum absorbances in the NIR range of 820–844 nm. Recent literature in NIR uncaging efforts using heptamethine cyanine dyes have reported photoactivation with 690–780 nm light (Gorka et al., 2014, 2015; Nani et al., 2015a, 2017). Our synthetic nanocage strategy allows us to improve upon this NIR window by successfully releasing the free TBI therapeutic, Gabapentin, from the cyanine nanocage after photoactivation with NIR light up to 850 nm. Our approach is supported by novel engineering technology that accelerates the photochemical activation that leads to the local release of the therapeutic drug being delivered to the brain. Given that the maximum absorbance of 850 nm is the ideal wavelength for penetrating through the skull and into the white matter, the NIR dyes within the range of 820–844 nm provide a therapeutically beneficial and efficient response (Yue and Humayun, 2015).

In order to enable the selective delivery of Gabapentin to TBI sites, NIR light (735–850 nm) irradiation of the cyanine moiety, in the presence of oxygen, leads to a rapid oxidative fragmentation of the cyanine dye (Gorka et al., 2014; Nani et al., 2015b). The chemical structure of the N,N-dimethylethylenediamine linker (green) is central to the photolytic breakdown of the cyanine dye and subsequent release of free drug cargo. Upon oxidative fragmentation of the cyanine moiety, the lone pair of electrons on the nitrogen atom connected to the cyanine can contribute to the formation of an iminium species, which is rapidly hydrolyzed in the biological aqueous environment. This allows for efficient and timely hydrolysis of the cyanine nanocage and release of the linker-drug moiety, which is then further fragmented to release the active drug (red). Notably, the fragmented cyanine and linker components are small molecules that can be readily excreted.

Overall, the local release of Gabapentin drug can be initiated and controlled through photoactivation using low energy NIR light. These compounds will be preferentially sequestered at the site of brain injury, where the BBB is compromised (Tran, 2014; Galgano et al., 2017), thereby allowing for TBI site-specific drug delivery, while minimizing off-target effects.

## Materials and Methods

### Cyanine Nanocage Synthesis

**Scheme 1:**
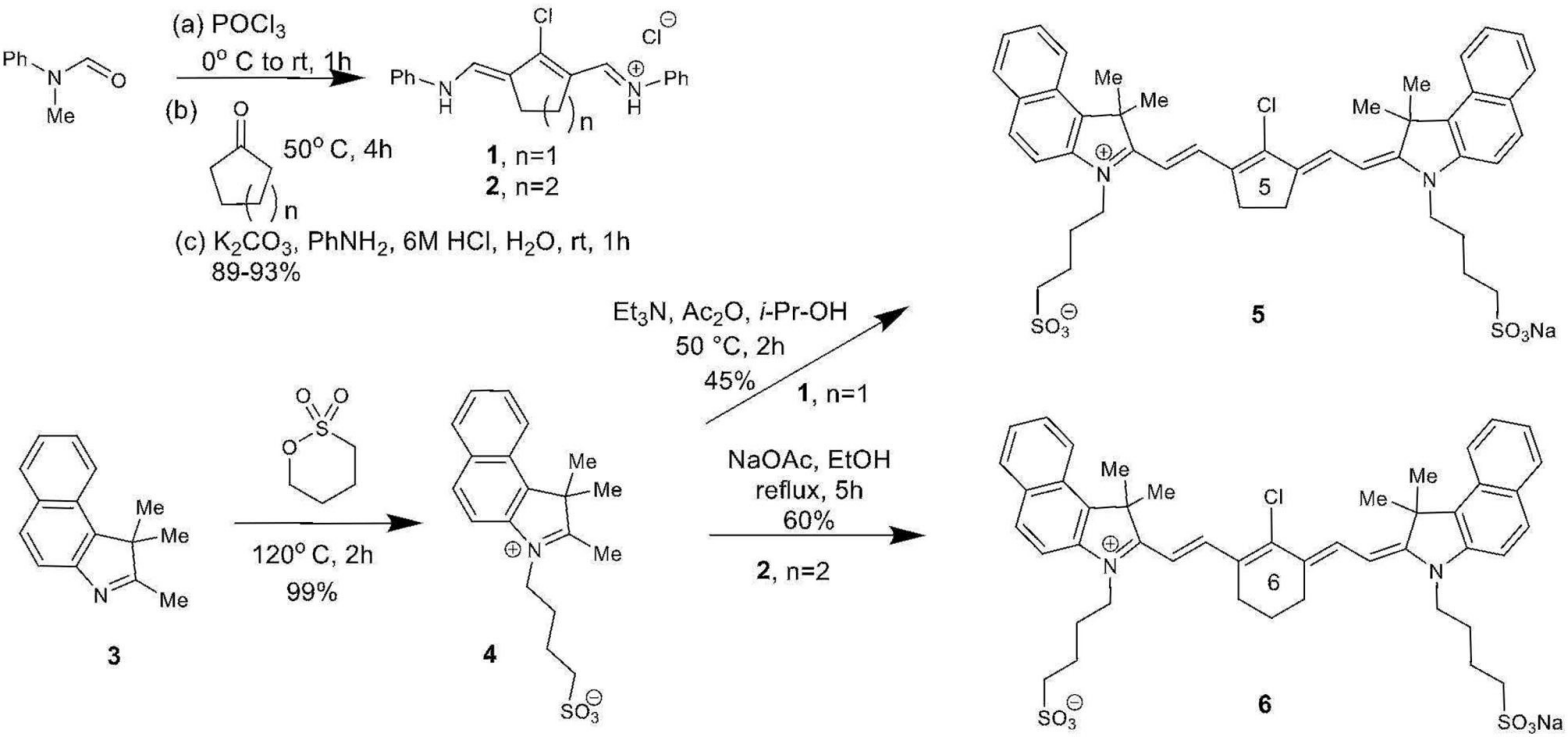
Total synthesis of chloro-substituted cyanine dyes **5** and **6**.

The total synthesis of two cyanine dyes of interest is shown in Scheme 1. Vilsmeier-Haack reaction with cyclopentanone and cyclohexanone formed the chlorinated di-aniline intermediates **1** (*n* = 1) and **2** (*n* = 2), while a simple alkylation of intermediate **3** gave the indolenium salt **4** in high yield (Nagao et al., 2007; Henary et al., 2009).

This reaction is tunable to the desired ring size in the final chloro-dye structure by starting with the respective cyclic ketone. Condensation of the indolenium salt **4** with the corresponding Vilsmeier-Haack reagents **1** and **2** gave the final cyanine dyes **5** and **6** under anhydrous conditions in ethanol in the presence of sodium acetate and acetic anhydride (Nagao et al., 2007; Henary et al., 2009). Cyanine dyes **5** and **6** absorb in the NIR range at 844 and 820 nm, respectively. Dye **5** containing the central cyclopentene unit has largely been unexplored as a cyanine nanocage for drug delivery and represents an exciting new development in this growing area of research.

**Scheme 2:**
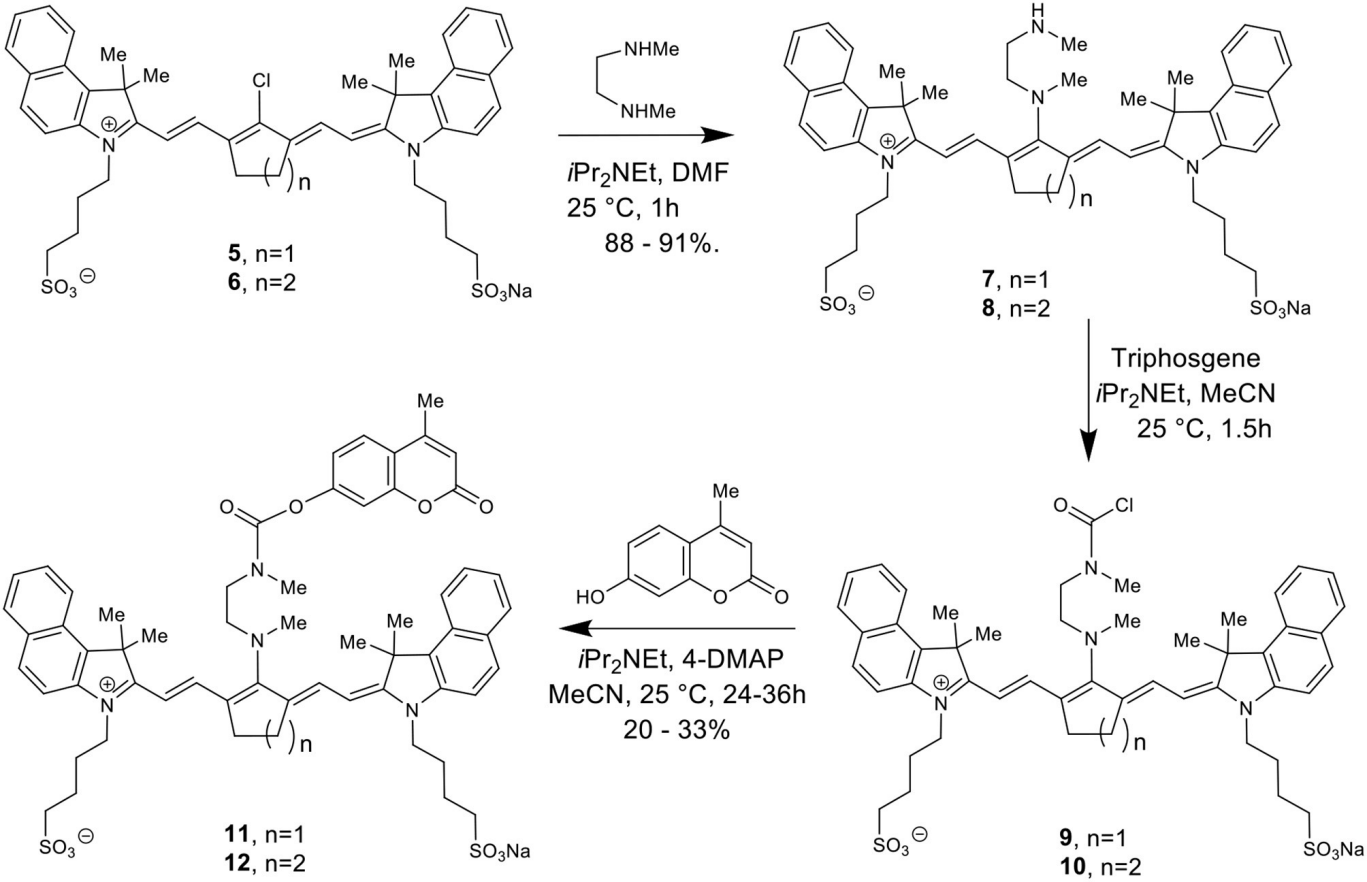
Total synthesis of cyanine-based caged coumarin derivatives **11** and **12**.

**Figure 2:**
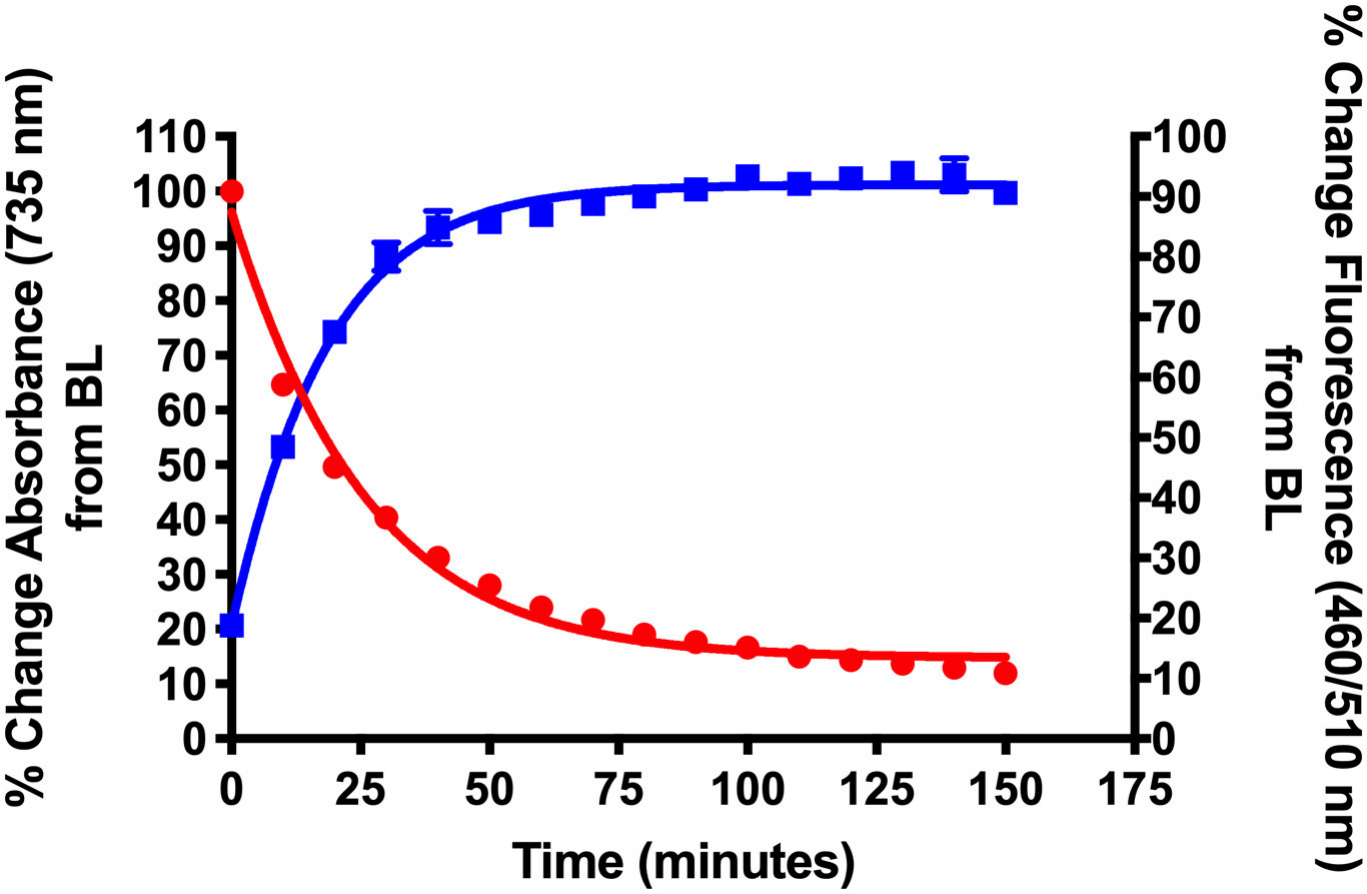
Validation of NIR light-activated drug delivery method using cyanine-based caged coumarin **12** (**BP114**). Photolysis efficiency and release of the 7-hydroxy-4-methyl coumarin cargo were shown.

Scheme 2 details the synthetic method used to couple phenol-containing cargo molecules to the dyes (Gorka et al., 2014). Upon formation of the dye-linker conjugates **7** and **8**, the amine linker can be activated under basic conditions using triphosgene to form the acid chloride intermediate. The phenolic cargo (7-hydroxy-4-methyl coumarin, 7-hydroxy-4-hydroxymethyl coumarin, 4-hydroxybenzyl alcohol) can then be added to the reaction under basic conditions with catalytic DMAP to form the final dye-cargo conjugates **11** and **12**. 7-Hydroxy-4-methyl coumarin was used as an initial proof-of-concept cargo molecule in order to facilitate easy measurement and quantification of cargo release during photolysis studies, shown in Figure 2.

### Synthesis of Cyanine Nanocages With Caged Gabapentin

Here we report the development of new synthetic methods for the formation of heptamethine cyanine dye-Gabapentin conjugates. The synthetic route was designed to conjugate Gabapentin through an additional carbamate linkage, akin to the method for coupling phenols shown in Scheme 2. The carbamate group has been widely used in drug discovery as an amide-ester hybrid mimicking a peptide bond (Ghosh and Brindisi, 2015). The ability of the carbamate moiety to form hydrogen bonds and interact with biological receptors may not only increase the cell permeability of our nanocage, but could also help promote the formation of drug release through interaction with enzymes in the brain.

**Scheme 3:**
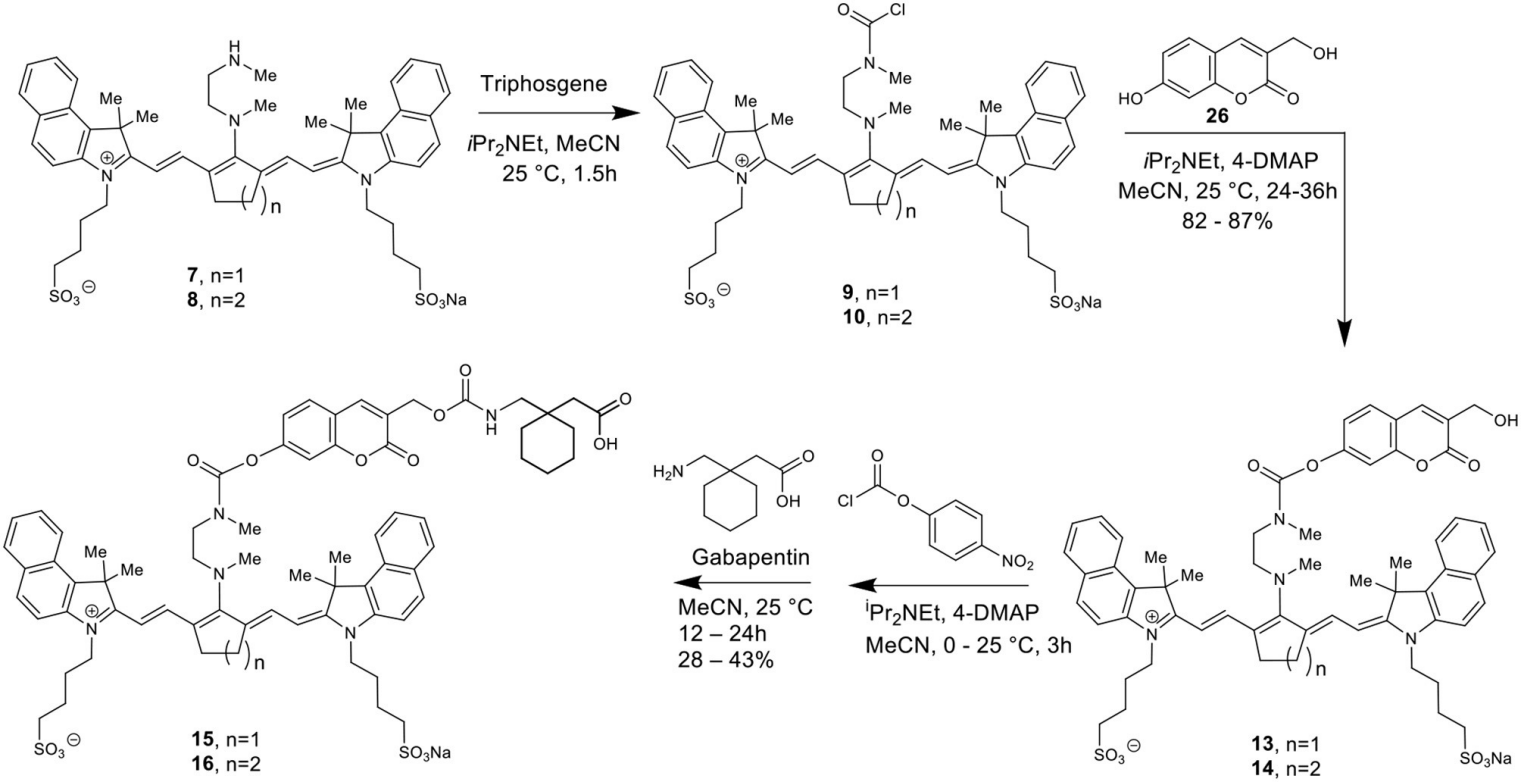
Total synthesis of cyanine-based caged Gabapentin derivatives **15** and **16**.

**Scheme 4:**
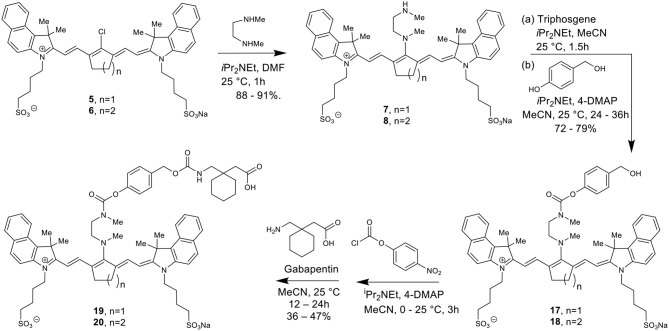
Total synthesis of cyanine-based caged Gabapentin derivatives **19** and **20**.

To make this carbamate coupling, 7-hydroxy-4-hydroxymethyl coumarin (**26**) and 4-hydroxybenzyl alcohol were used as linker components for the synthetic attachment of Gabapentin, as seen in Schemes 3, 4. Two different types of chemical linkers were used in order to form cyanine dye-drug conjugates with diverse capability of drug release quantification.

To form the final dye-drug conjugates, the hydroxy groups of compounds **13/14** and **17/18** were treated with 4-nitrophenyl chloroformate under basic conditions to afford the activated *p*-nitrophenyl carbonate intermediates. A Gabapentin solution in acetonitrile was then added directly to the reaction at room temperature to produce the final coupled products **15**/**16** and **19**/**20**.

## Results and Discussion

### Mechanism and Photolytic Efficiency of NIR Dye-Drug Conjugates

As a proof of concept, we developed an indocyanine-based nanocage unit for the NIR light-activated delivery of the Gabapentin drug in the brain. To do this, we must affirm that the dye-linker-drug compound has the physicochemical features required to quickly release the active drug. In this context, the breakdown of the parent compound must efficiently lead to the liberation of the drug cargo.

Synthesized cyanines and their respective conjugates were validated by NMR and LC-MS spectroscopy. In ^1^H NMR spectra, cyanine dyes (**5,6**) were characterized by chemical shifts corresponding to aromatic and vinyl protons (6.3–8.4 ppm) of the indolenium heterocycles and polyene chain, methyl protons (1.8–1.9 ppm) on the heterocycle, methylene protons (1.8–4.5 ppm) in the sulfonated N-butyl chains and methylene protons (1.8–3.0 ppm) in the central ring. Cyanine conjugates were also characterized according to their respective structures and caged cargo (diamine linker, 7-hydroxy-4-methyl coumarin, 7-hydroxy-4-hydroxymethyl coumarin, 4-hydroxybenzyl alcohol, and Gabapentin).

The synthetic NIR dye-cargo conjugates were tested in photolysis experiments to validate the drug delivery mechanism and determine the efficiency of cargo release upon NIR light activation for each specific dye and type of chemical linkage. The intact cyanine dye-cargo conjugates were solubilized in an aqueous solution at room temperature in the dark, with a NIR light LED (Thor Labs, continuous wavelength, 1,000 mA) positioned directly above the solution which was irradiated at 735 nm (M735L3) or 850 nm (M850L3).

Experiments were continuously exposed to light source for 120–180 min, where samples were collected at designated timepoints of 1, 5, 10, 30, 60, 90, 120, and 180 min. Samples were then analyzed using UV/Vis spectroscopy, fluorescence spectroscopy, and LC-MS to determine the extent of degradation of the parent compound and the formation of metabolites and intended cargo.

**Scheme 5:**
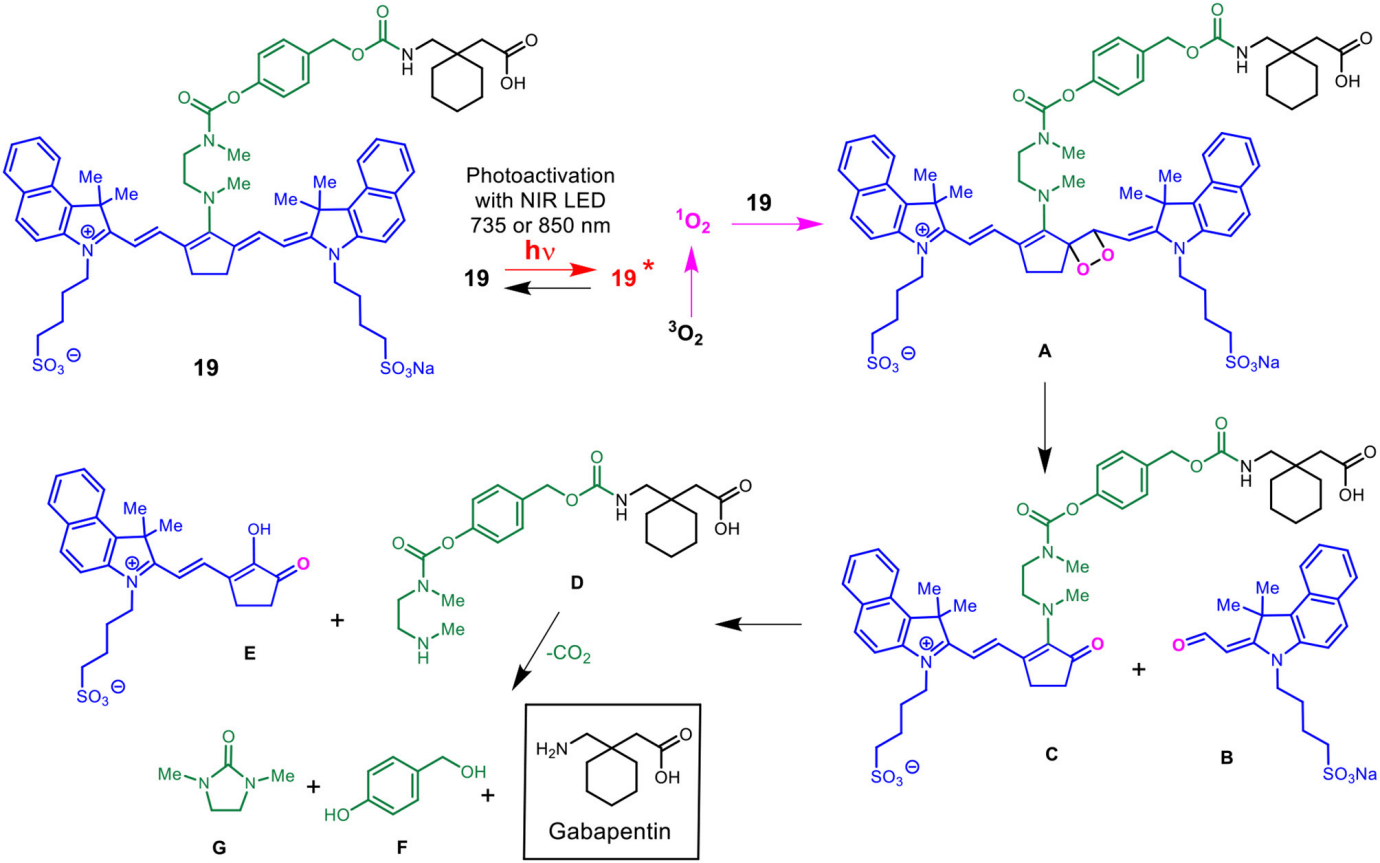
Mechanism for the NIR LED photoactivation, fragmentation and release of cyanine-based caged Gabapentin.

Scheme 5 details the proposed mechanism for the NIR light-activated degradation of the cyanine-Gabapentin conjugate **19**, leading to the fragmentation and breakdown of the cyanine dye and the release of free Gabapentin in its' active form. Photoactivation using NIR light (735/850 nm) irradiation in the presence of O_2_ leads to the excitation of the cyanine dye carrier and the activation of triplet oxygen ^**3**^O_**2**_ to its higher energy singlet oxygen form ^**1**^O_**2**_, which reacts with the cyanine dye to form dioxetane intermediate **A**.

The unstable oxetane intermediate **A** breaks down to aldehyde **B** and ketone **C**. Hydrolytic fragmentation of **C**, catalyzed by formation of an iminium precursor species, leads to the breakdown of the linker and the release of the linker-Gabapentin intermediate **D** together with the ketone intermediate **E**. Further fragmentation of **D** leads to the local release of free Gabapentin along with 4-hydroxymethyl phenol **F** and cyclic urea intermediate **G**.

Figure 2 shows the initial photolysis experiment conducted to validate our NIR light-based drug delivery method. Following the synthesis of the cyanine-based caged 7-hydroxy-4-methyl coumarin (**12**, **BP114**), it was irradiated with a NIR LED (735 nm) for 180 min. Samples were collected and analyzed for their % change in absorbance at 735 nm, the maximum absorbance wavelength of the intact starting material, and % change in fluorescence at λ_ex_ = 460 nm/λ_em_ = 510 nm, corresponding to 7-hydroxy-4-methyl coumarin.

We detected the breakdown of the caged coumarin (**12**, **BP114**) using UV/Vis monitoring at 735 nm and plotted over time. In addition, the production of 7-hydroxy-4-methyl coumarin over time was measured as a final % change of close to 100%. The loss of absorbance suggests that the parent cyanine dye undergoes light-mediated degradation, which subsequently induces the release of the free cargo, 7-hydroxy-4-methyl coumarin. The time where 50% of the parent compound broke down (T_50_) was ~22 min, which corresponded to 7-hydroxy-4-methyl coumarin release, as measured by fluorescence. These preliminary features suggest that these types of compounds have the efficiency to liberate adequate amount of active drug into the affected sites.

### Cellular Uptake of Cargo After NIR Activation

**Figure 3:**
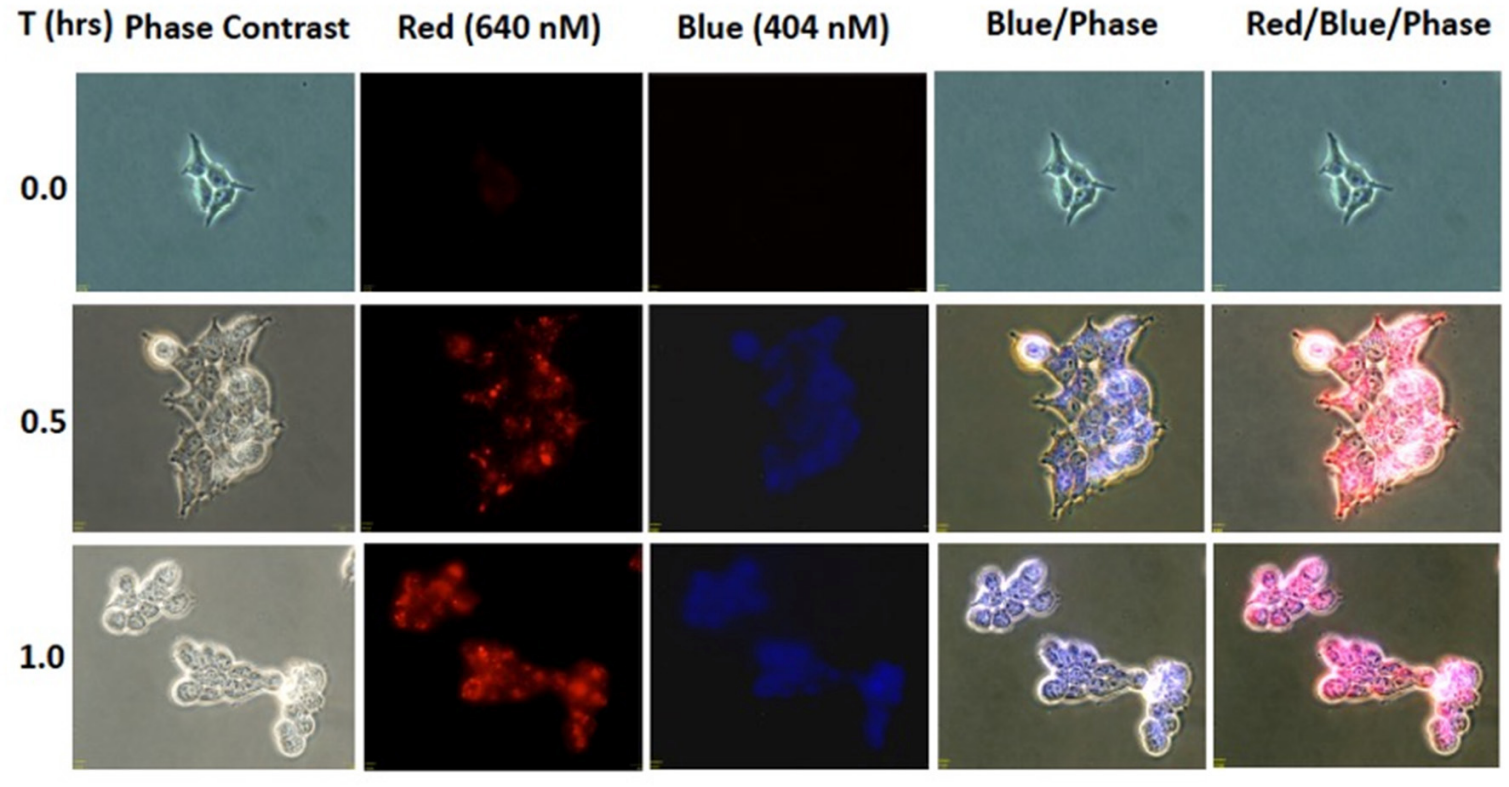
HEK293 cells were incubated with **BP114** containing caged coumarin. After 2 h of incubation, the cells were irradiated using 780 nm light for 0, 0.5, and 1 h. The uptake of coumarin was measured using 640 (red) and 404 (blue) nm.

To evaluate whether the NIR activation can uncouple the cargo, coumarin, to subsequently be taken up by the cells, HEK293 (0.3 × 10^6^ cells/well) was incubated in 50 μM **BP114** for 2 h. After incubation, the medium containing **BP114** was removed and fresh DMEM with 10% FBS was added. The cells were then irradiated using 780 nm wavelength for 0, 0.5, and 1 h, after which cells were then imaged using fluorescence microscopy at both 640 nm (red) and 404 nm (blue). Figure 3 summarizes the uptake of coumarin over the time course, where fluorescence was readily apparent after 0.5 and 1.0 h of LED illumination. These images are similar to coumarin given alone. No difference in fluorescence was detected between 0.5 and 1 h. These findings support our assertion that NIR activation can release the coumarin cargo that can be readily taken up by HEK293 cells.

**Figure 4:**
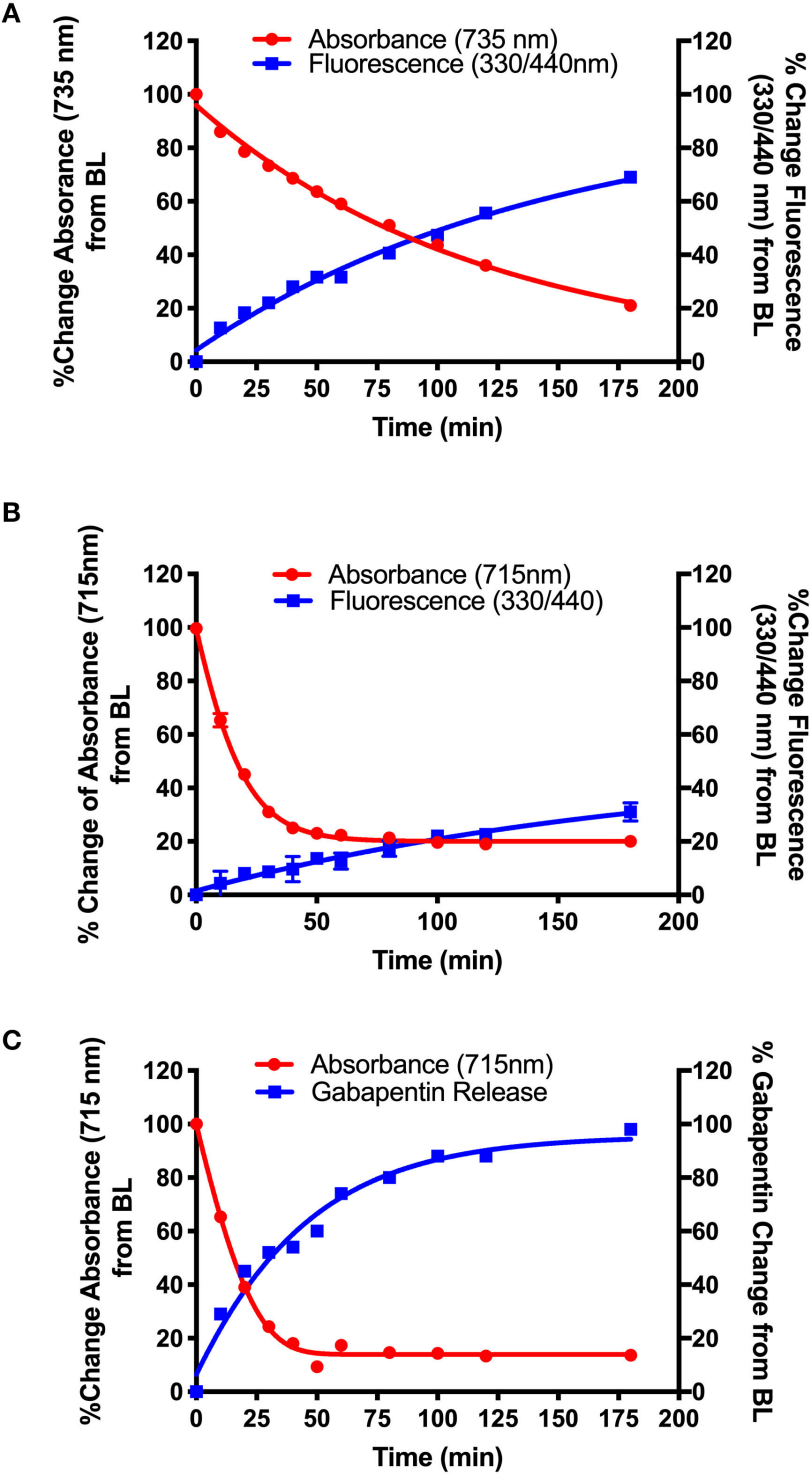
Photolysis efficiency of NIR cyanine dye-Gabapentin conjugates **(A) 16 (BP118)**, **(B) 15 (BP117)**, and **(C) 19 (BP116)**.

After validation of the uncaging strategy, NIR dye-Gabapentin conjugates were tested to determine their photolysis efficiency in an aerobic environment in the presence of NIR light (735 or 850 nm). Figure 4 shows photolysis data corresponding to NIR dye-Gabapentin conjugates **16** (**BP118**), **15** (**BP117**) and **19** (**BP116**). These compounds represent two different methods to monitor and quantify release of active drug. The difference between compounds **15–16** and compound **19** is the central ring connecting the cyanine dye-diamine linker to the caged Gabapentin. In **15–16**, Gabapentin is connected by 7-hydroxy-4-hydroxymethyl coumarin, allowing for the rapid monitoring of fluorescence to analyze drug release. Alternatively, Gabapentin is connected by 4-hydroxybenzyl alcohol in **19**, thus a more precise LC-MS measurement is used to analyze free drug release.

The synthetic design of **15–16** allows for the real-time analysis of drug release by measurement of the fluorescent modified-hydroxy coumarin linker, upon release of the modified coumarin-Gabapentin moiety from the photocage. Samples from the photolysis experiments of **15 (B)** and **16 (A)** were analyzed for % change in absorbance at 735 nm **(A)** and 715 nm **(B)**, the maximum absorbance of the starting material, and % change in fluorescence at λ_ex_ = 330 nm/λ_em_ = 440 nm, corresponding to the modified coumarin linker.

Figure 4 shows the results from the photolysis where the 5-member ring Gabapentin conjugate (**15**, **BP117**) in **B** is more efficiently broken down by 735 nm LED irradiation as compared to its 6-member ring analog (**16**, **BP118**) in **A**. The T_50_ is ~75 and 20 min for compounds **16** and **15**, respectively. These findings provide clear evidence for the photodegradation of **15** and **16** in a timely manner upon NIR irradiation. The data also highlights the importance of the central ring in the design. A possible factor contributing to the slower photoactivated breakdown of **16** as compared to **15** could be the formation of a more thermally stable dioxetane intermediate **A** (Scheme 5), resulting in a slower conversion to metabolites **B** and **C**.

While the formation of the cargo appeared to be inversely proportional to the breakdown of **BP118** in **A**, the release of the active cargo for **BP117 (B)** appeared to be slower, suggesting that an intermediate may be forming and thus slowing the liberation of the active cargo. Figure 4C, however, shows an inversely proportional relationship between the breakdown of parent compound **BP116** and formation of free Gabapentin, as measured by LC-MS, after 735 nm LED photoactivation. This analysis of Gabapentin release by LC-MS is very precise, as it measures only Gabapentin in solution. The T_50_ is ~20 min for this compound.

### Biological Evaluation of NIR Cyanine-Gabapentin Uncaging

Following initial photolysis studies of these NIR dye-Gabapentin conjugates, **19** (**BP116**) was chosen for further investigation in rat brain tissue to determine the uncaging process in biological tissues. We selected this compound as the lead candidate moving forward due to its' rapid photodegradation and release kinetics as observed during photolysis studies (Figure 4C). To this end, we developed an *ex vivo* method to study the light activated photolysis of **BP116**. Rat brain homogenates suspended in **BP116** solution were activated using a NIR LED source (850 nm) to initiate the photolysis process. The brain homogenate provided the potential enzymes that may further promote the release of the active cargo. To further simulate TBI, an 850 nm long pass cut-on filter was attached to the LED to eliminate wavelengths <850 nm to mimic the skull's ability to block shorter wavelengths. To monitor the formation of metabolites and end products, an aliquot of the samples was collected and analyzed at pre-defined time points.

To accomplish this, we developed a LC-MS based targeted metabolomics assay to characterize and quantify each metabolite found after photoactivation. Metabolites were identified using an untargeted approach, where the molecular weights and daughter ions were used to define the structures. This information allowed us to develop a targeted approach, where the level of the metabolites could be quantified using multiple reaction monitoring (MRM).

**Scheme 6:**
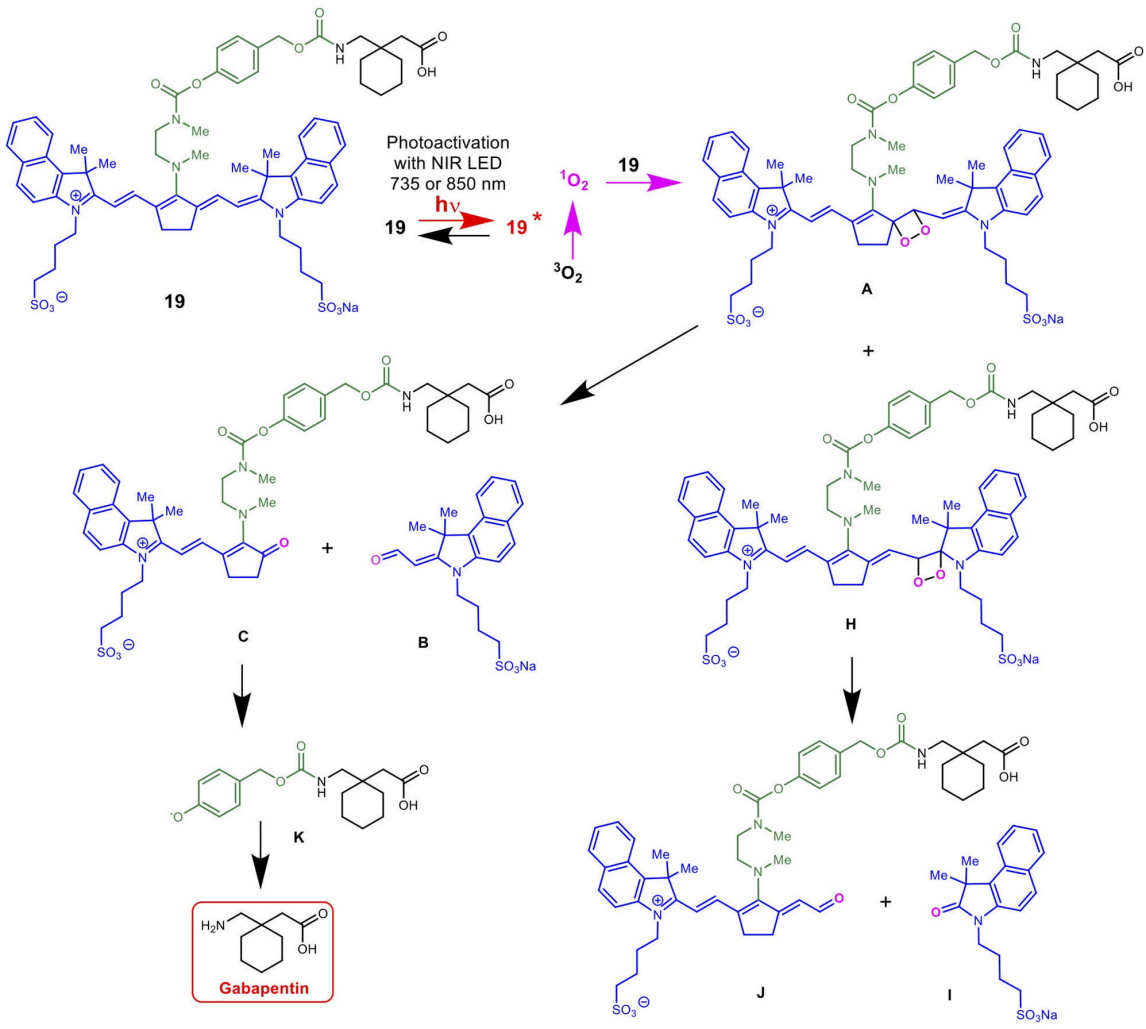
Mechanism for the NIR LED-activated breakdown of **19** (**BP116**) in rat brain homogenates as determined by targeted LC-MS, leading to the delivery of Gabapentin.

**Figure 5:**
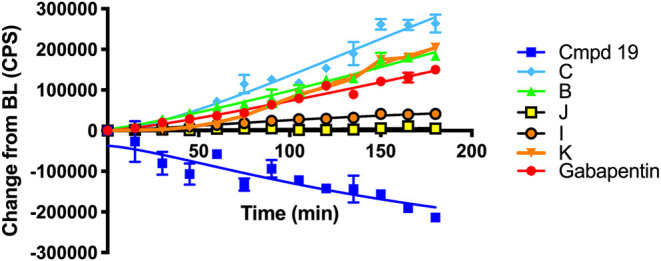
Baseline changes of **BP116** breakdown and formation of metabolites over 180 min in rat brain homogenates upon NIR LED (850 nm) photoactivation.

Scheme 6 details the mechanistic breakdown pathway based on the products observed in the rat brain homogenates after NIR irradiation at 850 nm. The result of this time course study is summarized in Figure 5, which monitors the levels of **BP116** and the formation of metabolites over time. The level of each analyte abundance was measure in counts per second (CPS). In this context, **BP116** breakdown over time led to the production of two key photo-oxidized metabolite compounds: **C**, resulting from dioxetane intermediate **A**; and **J**, resulting from dioxetane intermediate **H** (Scheme 6).

As observed in Figure 5, production of photo-oxidized metabolite compound **C** corresponded to the formation of aldehyde **B**, while the production of compound **J** corresponded to formation of ketone **I**. This study suggests that the major photolysis products were compound **B** and **C**, where compounds **I** and **J** were minor breakdown products. This study showed that formation of **C** was the rate limiting step in release of free Gabapentin. Interestingly, the formation of HBA-Gabapentin, compound **K**, was a product of **C** breakdown. The formation of Gabapentin is parallel to the production of compound **K**, which further supports our hypothesis that once the linker-Gabapentin is produced, it will spontaneously breakdown to release the Gabapentin cargo.

Regression analysis between compounds **C** and **J** formation was correlated with **BP116** breakdown, where the *R*^2^ were 0.7943 vs. 0.2603, respectively. We further correlated the formation of metabolite **C** with formation of **K**, where a near linear *R*^2^ = 0.958 further verified that these metabolites are central to the metabolic pathways.

These findings were also verified in the plasma of animals receiving **BP116**. The breakdown of **BP116** following LED exposure had a long lag time (~20–40 min). Once compound **C** was formed, the conversion to compound **K** and Gabapentin was incremental and thus difficult to detect in *in vivo* experiments.

In summary, the key finding of this study is that NIR light-activated breakdown of **BP116** led to the formation of key metabolite **C**. Compound **C** is a relatively stable product that required ~45 min of light activation to generate adequate levels of compound **K**, the linker-Gabapentin complex, which spontaneously formed Gabapentin. Based on this study, efforts to improve the cyanine nanocage platform are ongoing, in order to circumvent the rate-limiting step and enhance Gabapentin delivery to the brain tissues.

The data presented suggest that the concept of NIR-activation of photocages for the purpose of drug delivery is a viable strategy. We have shown that the cargo can be released after the NIR light activated breakdown of the parent compound. This report suggests that the structure of the nanocage, in particular the central ring, is an important factor affecting the rate of photolysis and the formation of the active cargo.

The chemical linkage between the cyanine dye and the TBI therapeutic, Gabapentin, can also be modified to incorporate different methods of analysis of drug release. Finally, we have shown the NIR light-activated (850 nm) uncaging and release of Gabapentin in our novel brain homogenate system using targeted and untargeted LC-MS methods. These studies further serve to guide our development of heptamethine cyanine nanocages for the targeted delivery of Gabapentin to TBI sites.

## Data Availability Statement

The raw data supporting the conclusions of this article will be made available by the authors, without undue reservation.

## Ethics Statement

The animal study was reviewed and approved by University of Southern California Institutional Animal Care and Use Committee.

## Author Contributions

The manuscript was written with contributions of all authors. All authors have approved the final version of the manuscript.

## Conflict of Interest

The authors declare that the research was conducted in the absence of any commercial or financial relationships that could be construed as a potential conflict of interest.
