# Supplementary material for: Cyanine Nanocages Activated by Near-Infrared Light for the Targeted Treatment of Traumatic Brain Injury
Source: Front Chem. 2020 Aug 31;8:769. doi: 10.3389/fchem.2020.00769 (PMC7489144; doi:10.3389/fchem.2020.00769)
Supplement: Supplementary file 1 [file Data_Sheet_1.pdf]

Supplementary Material

1 Experimental Procedures and NMR Data

General Materials and Methods: Unless otherwise stated, all reactions were conducted in oven-dried glassware under an atmosphere of argon using anhydrous solvents. All commercially obtained reagents were used as received. Flash chromatography was performed using Biotage SNAP Ultra C18 columns (12g and 30g) on the Biotage Isolera One instrument. LC-MS analyses were conducted on an Agilent 1260 Infinity ESI-MS using the following method: 30-100% MeCN (90% MeCN, 10% H_2O) in H_2O with 0.1% formic acid, 0.300 mL/min, 14 min. All NIR dye compounds were observed in the mass spectral data as the parent molecule without the Na^+ counterion. NMR spectra was recorded on Varian 400, 500 or 600 MHz NMR spectrometers using the stated deuterated solvents.

1.1 Synthesis of NIR Cyanine Dyes

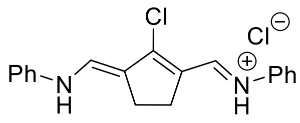

1

1: To a solution of N-methylformanilide (6.16 mL, 49.94 mmol) in chloroform (6.5 mL) was added phosphorous oxychloride (7.0 mL, 74.87 mmol) slowly at 0° C. After complete addition, the reaction mixture was stirred at room temperature for 1 hr. Cyclopentanone (1.77 mL, 19.97 mmol) was then added and the reaction was heated at 50° C for 4 hr. The mixture was cooled to room temperature, and the following reagents were added: potassium carbonate (5.0 g), then aniline (4.12 mL, 45.10 mmol), 6N HCl (3.75 mL), H_2O (25 mL). After addition, the mixture was stirred at room temperature for 1 hr. DCM (25 mL) was added to the reaction mixture and let stand for a while. Finally, the solid was filtered, washed with acetone and H_2O, and dried overnight in a vacuum oven to afford the light purple solid (6.44 g, 93%). **^{1}H NMR** ($(CD_3)_2SO$, 600 MHz): δ 8.31 (s, 1H), 7.62 (d, *J* = 8.0 Hz, 2H), 7.57 – 7.40 (m, 4H), 7.32 – 7.18 (m, 4H), 6.91 (s, 1H), 2.75 (s, 4H). **MW** (without Cl^- anion): 309.82 g/mol.

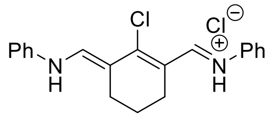

2

2: N,N'-Dimethylformamide (3.7mL, 48mmol) was placed in an oven dried 100mL round bottom flask, placed under Argon, and cooled to 0°C. The DMF was charged dropwise with phosphorus oxychloride (3.22mL, 34.5mmol) and stirred for 30 minutes at 0°C. Cyclohexanone (1.56mL, 15mmol) was added and the reaction mixture was refluxed for 1 hour. After cooling to room temperature, the flask was charged dropwise with 50mL of a 1:1 mixture of aniline and ethanol and stirred at room temperature for 30 minutes. The red solution was then poured into a flask with cold 2M HCl(aq) and was refrigerated for several hours. A purple solid was collected by vacuum filtration and washed with ice cold water. The solid was dried for several hours in a vacuum oven to yield a dark purple powdery solid (4.322g, 89%). **^{1}H NMR** (400 MHz, DMSO-d_6) δ 8.54 (s, 1H), 7.64 – 7.58 (m, 4H), 7.49 – 7.44 (m, 4H), 7.35 – 7.25 (m, 3H), 2.75 (t, *J* = 6.2 Hz, 4H), 1.86 (p, *J* = 6.1 Hz, 2H). **MW** (without Cl^- anion): 323.84 g/mol.

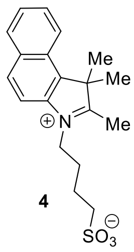

4

4: 1,1,2-trimethyl-1H-benzo[e]indole (1.01 g, 4.83 mmol) and 1,4-butane sultone (1.48 mL, 14.48 mmol) were added neat to a round-bottom flask at normal atmosphere, and stirred for 2 hr at 120° C. After 2 hr, the solid reaction mixture was washed thoroughly with acetone, filtered, and dried in a vacuum oven to afford the

grey-blue solid product (1.65 g, 99%). **^{1}H NMR** (($(CD_3)_2SO$, 600 MHz): δ 8.36 (d, *J* =8.3 Hz, 1H), 8.28 (d, *J* =8.9 Hz, 1H), 8.23-8.19 (m, 2H), 7.80-7.76 (m, 1H), 7.74-7.70 (m, 1H), 4.61 (t, *J* = 7.9 Hz, 2H), 2.95 (s, 3H), 2.54-2.52 (m, 2H), 2.08-2.01 (m, 2H), 1.81-1.76 (m, 2H), 1.76 (s, 6H). **MW:** 345.46 g/mol.

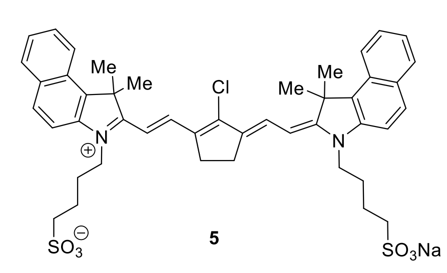

5: Indolenium salt **4** (1.50 g, 4.34 mmol) and dianiline **1** (0.5 g, 1.45 mmol) were dissolved in isopropyl alcohol (7 mL) in a round-bottom flask. To the reaction mixture was added triethylamine (1.21 mL) then acetic anhydride (0.41 mL). The reaction was stirred at 50 °C for 2 hr, while monitored by LC-MS. Upon completion, the reaction was cooled to room temperature, added to diethyl ether, and cooled at 2° C to afford crystallization of the product. Product was purified by recrystallization from MeOH/Et_2O, followed by reverse-phase chromatography (30-100% ACN in H_2O) to afford **5** as a green solid (0.54 g, 45%). **LC-MS analysis**: λ_{max} = 844 nm, MS: [M+H] 813; [M-H] 811 (MW of **5**: 835.45 g/mol). **^{1}H NMR** (($(CD_3)_2SO$, 600 MHz): δ 8.27 (d, *J* = 8.6 Hz, 2H), 8.09-8.05 (m, 4H), 7.86 (d, *J* = 12.9 Hz, 2H), 7.80 (d, *J* = 8.8 Hz, 2H), 7.67-7.62 (m, 2H), 7.54-7.47 (m, 2H), 6.25 (d, *J* = 14.2 Hz, 2H), 4.33 (t, *J* = 7.6 Hz, 4H), 3.15-3.05 (m, 4H), 3.01 (s, 4H), 1.94 (s, 12H), 1.89 (p, *J* = 7.8, 7.4 Hz, 4H), 1.82-1.74 (m, 4H).

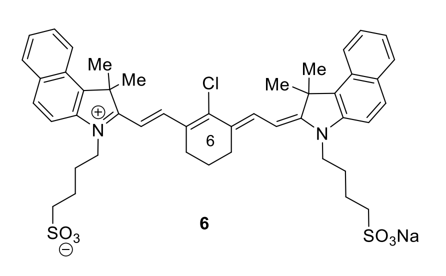

6: Indolenium salt **4** (1.108g, 3.206mmol), dianiline **2** (0.259g 0.802mmol), and sodium acetate (0.1604g, 1.96mmol) were combined in an oven-dried 50mL flask and placed under Argon. The reagents were dissolved in 200 proof ethanol (10mL) and heated to reflux for 5 hours. The now blue-green solution was cooled to room temperature and concentrated under reduced pressure. The residue was dry loaded onto celite and purified by reverse phase column chromatography on a C18 column with the product eluting in 60% acetonitrile in water. The relevant fractions were combined and concentrated under reduced pressure and transferred to an 8-dram vial with a minimal amount of DMF. The vial was filled with ethyl ether (25mL) and cooled in a refrigerator overnight. The vial was rewarmed to room temperature, and the top ether layer was removed with a pipette while leaving the crystallized solid at the bottom of the container. More fresh ether was added to the container with the green solid, and the solid was allowed to settle to the bottom. The ether layer was removed again once the solid settled to the bottom of the container. Subsequent washing with ether and removal of the ether was performed until the ether layer is clear. The solid was dried under a constant stream of dry nitrogen to afford IR-820 as a dark green powdery solid (0.396g, 60%). Drying the solid was only performed directly before usage of the compound. The product was stored in an 8-dram vial filled with ether to separate the solid at the bottom from the moisture and oxygen in the air. **LC-MS analysis**: λ_{max} (820 nm), observed [M+(-H)] = 827 [M-(-H)] = 825. **^{1}H NMR** (500 MHz, DMSO-d_6) δ 8.41 – 8.34 (m, 2H), 8.30 (d, *J* = 8.7 Hz, 2H), 8.12 – 8.03 (m, 4H), 7.86 (d, *J* = 9.1 Hz, 2H), 7.68 – 7.62 (m, 2H), 7.52 (t, *J* = 8.4, 6.9 Hz, 2H), 6.56 (d, *J* = 14.2 Hz, 2H), 4.51 (t, *J* = 7.9 Hz, 4H), 2.79 (t, *J* = 6.3 Hz, 4H), 2.61 (t, *J* = 6.7 Hz, 4H), 2.44 – 2.37 (m, 2H), 2.13 – 2.03 (m, 4H), 1.96 (s, 12H), 1.91 – 1.72 (m, 4H).

1.2 Coupling of Linker to NIR Cyanine Dyes

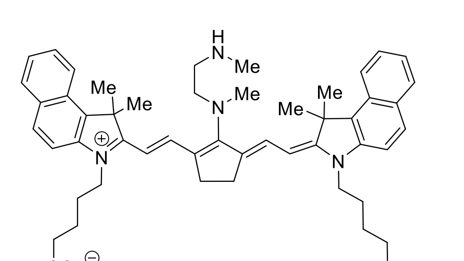

7

7: To a solution of **5** (0.121 g, 0.145 mmol) in DMF (10 mL) in an 8-dram vial under normal atmosphere was added N,N'-diisopropylethylamine (0.076 mL, 0.434 mmol) and N,N'-dimethylethylenediamine (0.047 mL, 0.434 mmol). The reaction mixture was stirred at room temperature for one hour, and LC-MS analysis confirmed the complete conversion of **5** to **7**. Reaction mixture was added to ether and cooled at 2° C for several hours to afford crystallization of the product. The product was purified by washing with 3 x 25 mL diethyl ether and removing the top ether layer from the solid after each wash. After the third wash, the solid was dried under a flow of N_2 to afford **7** as a blue solid (0.112 g, 88%). **LC-MS analysis**: λ_{max} = 738 nm, MS: [M+H] 865; [M-H] 863 (MW of **7**: 887.14 g/mol). **^{1}H NMR** ($(CD_3)_2SO$, 600 MHz): δ 8.15 (d, *J* = 8.7 Hz, 2H), 7.99 (d, *J* = 8.6 Hz, 4H), 7.88-7.84 (m, 2H), 7.63 (d, *J* = 8.8 Hz, 2H), 7.61-7.56 (m, 2H), 7.43-7.39 (m, 2H), 5.80 (d, *J* = 12.9 Hz, 2H), 4.18-4.12 (m, 4H), 3.93-3.88 (m, 3H), 3.07-3.01 (m, 4H), 2.86 (s, 2H), 2.81-2.76 (m, 4H), 2.56 (s, 2H), 2.43 (s, 3H), 1.88 (s, 12H), 1.77-1.72 (m, 8H).

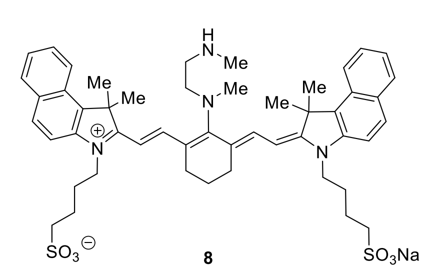

8

8: To a solution of **6** (3.3, 0.0473 g, 0.0560 mmol) in DMF (4 mL) in an 8-dram vial was added N,N'-Diisopropylethylamine (0.030 mL, 0.167 mmol) and N,N'-dimethylethylenediamine (0.018 mL, 0.167 mmol). The reaction mixture was stirred at room temperature for 80 minutes, and the completion of reaction was confirmed by LC/MS. The reaction mixture was charged with approximately 25mL of ethyl ether, filling the 8-dram vial, and was cooled to 4°C for several hours. The vial was rewarmed to room temperature, and the top ether layer was removed with a pipette while leaving the crystallized solid at the bottom of the container. More fresh ether was added to the container with the blue solid, and the solid was allowed to settle to the bottom. The ether layer was removed again once the blue solid settled to the bottom of the container. Subsequent washing with ether and removal of the ether was performed until the ether layer is clear. The solid was dried under a constant stream of dry nitrogen to afford **8** as a dark blue solid (0.039 g, 91%, correcting for IR-820 because it contains 80% dye). Drying the solid was only performed directly before usage of the compound. The product was stored in an 8-dram vial filled with ether to separate the blue solid at the bottom from the moisture and oxygen in the air. **LC-MS analysis**: λ_{max} (770 nm), observed [M+(-H)] = 879 [M-(-H)] = 877. **MW** of **8:** 901.17 g/mol.

1.3 General Procedure for the Synthesis of Dyes 11-12, 13-14 and Gabapentin Conjugates 15-16

11-12, 13-14: To a solution of dye-linker (**7, 8**) (0.100 mmol) in acetonitrile (7 mL) was added N,N'-diisopropylethylamine (0.052 mL, 0.301 mmol). Triphosgene in acetonitrile (0.015 g, 0.050 mmol) was added dropwise over several minutes, and reaction mixture was stirred at room temperature for 1.5 hours. LC-MS analysis confirmed the complete conversion of dye-linker to the activated acid chloride after 1.5 hours. To the reaction mixture containing the newly formed acid chloride was added N, N'-diisopropylethylamine (0.052 mL, 0.301 mmol), respective alcohol (0.301 mmol), and 4-dimethylaminopyridine (0.006 g, 0.050 mmol). The reaction was stirred at room temperature for 24-48 hr, until LC-MS analysis confirmed the disappearance of the acid chloride and product formation. Mixture was added to ether and cooled at 2° C to afford crystallization of the product. The product was purified by washing with 3 x 25 mL diethyl ether and removing the top ether layer from the solid after each wash. Compounds were subsequently purified by reverse-phase chromatography

(30-100% ACN in H_2O). Both products were dried under a flow of N_2 to afford **11-12** and **13-14** as blue solids (33-87%).

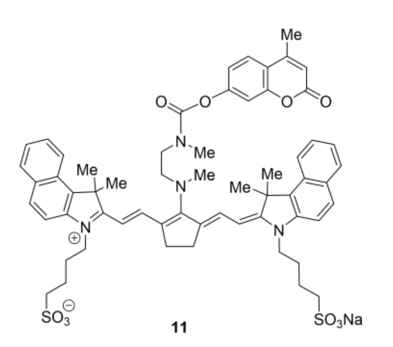

11

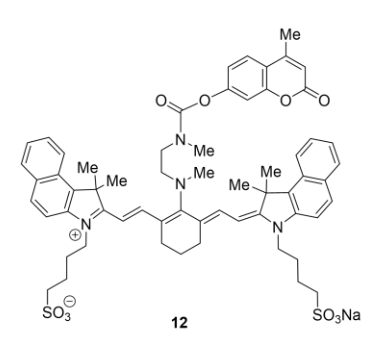

12

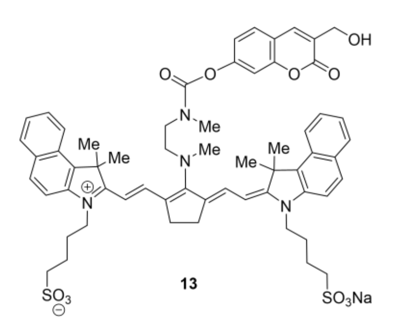

13

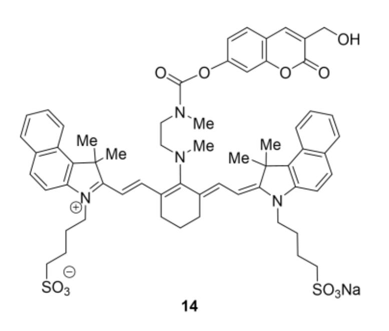

14

11: Synthesized with **7** and 4-methylumbelliferone according to the general procedure outlined above to afford solid blue product (0.015 g, 42%). **LC-MS Analysis**: λ_{max} = 715 nm, MS: [M+H] 1067; [M-H] 1065 (**MW**: 1089.3 g/mol). **^{1}H NMR** (600 MHz, DMSO-d_6): δ 8.18-8.12 (m, 2H), 8.01-7.96 (m, 6H), 7.59 (t, *J* = 9.3 Hz, 3H), 7.53 (t, *J* = 7.7 Hz, 2H), 7.44-7.36 (m, 4H), 6.52 (s, 1H), 5.81 (s, 2H), 4.15-4.05 (m, 4H), 3.64-3.56 (m, 4H), 3.17-3.16 (m, 6H), 2.99 (m, 2H), 2.82-2.78 (m, 5H), 2.73 (s, 4H), 1.89-1.87 (m, 8H), 1.76 (s, 12H).

12: Synthesized with **8** and 4-methylumbelliferone according to the general procedure outline above to afford solid blue product (0.010 g, 33%). **LC-MS Analysis**: λ_{max} = 738 nm, MS: [M+H] 1081; [M-H] 1079 (**MW**: 1103.3 g/mol). **^{1}H NMR** (600 MHz, Methanol-d_4) δ 8.11 (d, *J* = 9.0 Hz, 2H), 7.97-7.90 (m, 4H), 7.81 (s, 2H), 7.69 (d, *J* = 8.6 Hz, 1H), 7.59-7.47 (m, 5H), 7.45-7.33 (m, 3H), 7.15-7.08 (m, 1H), 6.06 (s, 2H), 4.23-4.15 (m, 4H), 3.65 (s, 2H), 3.55 (s, 1H), 3.40 (d, *J* = 8.8 Hz, 2H), 3.17 (s, 1H), 3.10 (s, 3H), 2.90 (t, *J* = 7.3 Hz, 6H), 2.62 (s, 4H), 2.46 (s, 1H), 2.31 (s, 3H), 2.07-1.98 (m, 8H), 1.90 (s, 12H).

13: Synthesized with **7** and hydroxy-coumarin **26** according to the general procedure outlined above to afford solid blue product (0.087 g, 87%). **LC-MS Analysis**: λ_{max} = 720 nm, MS: [M+H] 1083; [M-H] 1081 (**MW**: 1105.3 g/mol).

14: Synthesized with **8** and hydroxy-coumarin **26** according to the general procedure outline above to afford solid blue product (0.0929 g, 82%). **LC-MS Analysis**: λ_{max} = 743 nm, MS: [M+H] 1097; [M-H] 1095 (**MW**: 1119.3 g/mol).

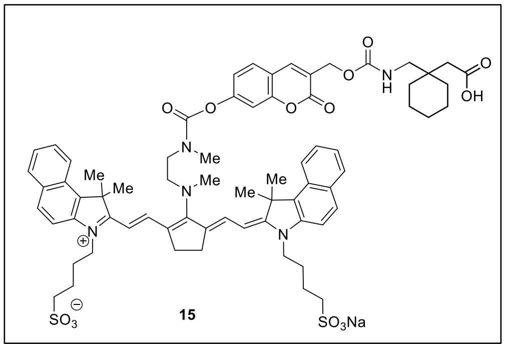

15

15-16: To a solution of **13** or **14** (0.114 mmol) in acetonitrile (8 mL) was added N,N'-diisopropylethylamine (0.099 mL, 0.569 mmol) and 4-dimethylaminopyridine (0.014 g, 0.114 mmol). 4-nitrophenyl chloroformate (0.057 g, 0.285 mmol) in acetonitrile was added dropwise to reaction mixture at 0° C over 5-10 min. Reaction was warmed up to room temperature and stirred for 1.5-5 hr, until LC-MS analysis confirmed the disappearance of starting material and formation of the activated carbonate. Gabapentin (0.058 g, 0.342 mmol) in acetonitrile was then

added to the reaction mixture and stirred at room temperature for 12-24 hr until LC-MS analysis confirmed the disappearance of activated carbonate and formation of the desired product. The reaction mixture was filtered to remove excess Gabapentin and then filtrate added to ether and cooled at 2° C to afford crystallization of the product. The product was purified by washing with 3 x 25 mL diethyl ether and removing the top ether layer from the solid after each wash. Compounds were subsequently purified by reverse-phase chromatography (30-100% ACN in H_2O). Both products were dried under a flow of N_2 to afford **15-16** as blue solids (28-43%).

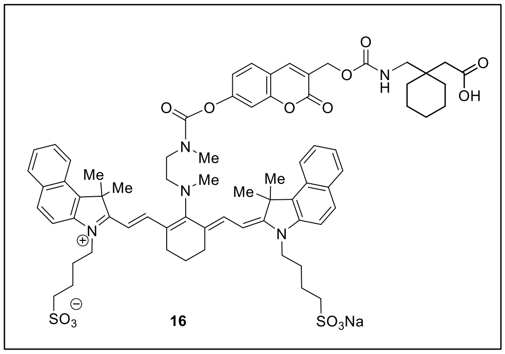

15: Synthesized with **13** according to the general procedure outline above to afford solid blue product (0.045 g, 43%). **LC-MS Analysis**: λ_{max} = 715 nm, MS: [M-H (+Na^+)] 1301 (**MW**: 1302.5 g/mol).

16: Synthesized with **14** according to the general procedure outline above to afford solid blue product (0.042 g, 28%). **LC-MS Analysis**: λ_{max} = 736 nm, MS: [M+H (+Na^+)] 1316; [M-H (+Na^+)] 1314 (**MW**: 1316.6 g/mol).

1.4 General Procedure for Synthesis of NIR Dyes 17-18 and Gabapentin Conjugates 19-20

17-18: To a solution of dye-linker (**7, 8**) (0.100 mmol) in acetonitrile (7 mL) was added N,N'-diisopropylethylamine (0.052 mL, 0.301 mmol). Triphosgene in acetonitrile (0.015 g, 0.050 mmol) was added dropwise over several minutes, and reaction mixture was stirred at room temperature for 1.5 hours. LC-MS analysis confirmed the complete conversion of dye-linker to the activated acid chloride after 1.5 hours. To the reaction mixture containing the newly formed acid chloride was added N, N'-diisopropylethylamine (0.052 mL, 0.301 mmol), 4-hydroxybenzyl alcohol (0.301 mmol), and 4-dimethylaminopyridine (0.006 g, 0.050 mmol). The reaction was stirred at room temperature for 24-48 hr, until LC-MS analysis confirmed the disappearance of the acid chloride and product formation. Mixture was added to ether and cooled at 2° C to afford crystallization of the product. The product was purified by washing with 3 x 25 mL diethyl ether and removing the top ether layer from the solid after each wash. Compounds were subsequently purified by reverse-phase chromatography (30-100% ACN in H_2O). Both products were dried under a flow of N_2 to afford **17-18** as blue solids (72-79%).

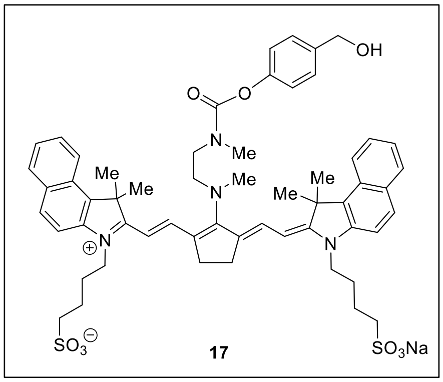

17: Synthesized with **7** according to the general procedure outlined above to afford solid blue product (0.075 g, 72%). **LC-MS Analysis**: λ_{max} = 718 nm, MS: [M+H] 1015; [M-H] 1013 (**MW**: 1037.3 g/mol). **^{1}H NMR** (600 MHz, Methanol-*d*$_4$): δ 8.20 (d, J = 8.2 Hz, 1H), 8.01-7.94 (m, 5H), 7.77 (d, J = 8.8 Hz, 1H), 7.56 (d, J = 8.7 Hz, 3H), 7.47-7.44 (m, 3H), 7.38-7.35 (m, 3H), 7.25 (d, J = 8.7 Hz, 1H), 6.95-6.92 (m, 1H), 6.14 (d, J = 13.7 Hz, 2H), 4.26-4.23 (m, 2H), 4.10 (m, 4H), 3.86-3.82 (m, 2H), 3.69 (s, 2H), 3.57 (s, 2H), 3.14-3.10 (m, 2H), 3.05-2.99 (m, 3H), 2.81 (s, 3H), 2.77-2.74 (m, 2H), 2.61-2.55 (m, 2H), 1.95-1.91 (m, 8H), 1.88-1.78 (m, 12H).

18: Synthesized with **8** according to the general procedure outlined above to afford solid blue product (0.093 g, 79%). **LC-MS Analysis**: λ_{max} = 736 nm, MS: [M+H] 1029; [M-H] 1027 (**MW**: 1051.3 g/mol). **^{1}H NMR** (600 MHz, DMSO-*d*$_6$) δ 8.16 – 8.10 (m, 1H), 8.07 (d, *J* = 8.6 Hz, 1H), 8.02 – 7.97

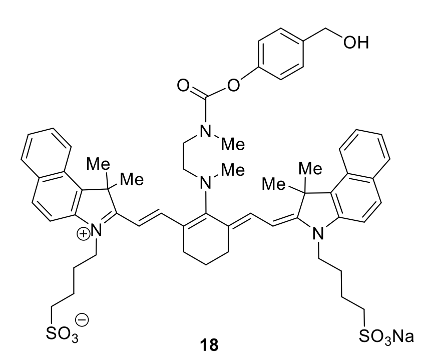

18

(m, 2H), 7.95 (s, 1H), 7.86 – 7.78 (m, 3H), 7.68 – 7.54 (m, 5H), 7.49 (t, *J* = 7.6 Hz, 2H), 7.45 – 7.39 (m, 1H), 7.35 (t, *J* = 7.5 Hz, 2H), 6.05 – 5.93 (m, 2H), 4.51 – 4.33 (m, 2H), 4.24 – 4.06 (m, 5H), 3.96 (s, 1H), 3.83 – 3.69 (m, 3H), 3.17 (s, 1H), 3.06 (s, 1H), 2.98 (s, 1H), 2.81 – 2.69 (m, 3H), 2.56 (s, 2H), 1.98 – 1.77 (m, 13H), 1.75 (s, 12H).

19-20: To a solution of **17** or **18** (0.114 mmol) in acetonitrile (8 mL) was added N,N'-diisopropylethylamine (0.099 mL, 0.569 mmol) and 4-dimethylaminopyridine (0.014 g, 0.114 mmol). 4-nitrophenyl chloroformate (0.057 g, 0.285 mmol) in acetonitrile was added dropwise to reaction mixture at 0° C over 5-10 min. Reaction was warmed up to room temperature and stirred for 1.5-5 hr, until LC-MS analysis confirmed the disappearance of starting material and formation of the activated carbonate. Gabapentin (0.058 g, 0.342 mmol) in acetonitrile was then added to the reaction mixture and stirred at room temperature for 12-24 hr until LC-MS analysis confirmed the disappearance of activated carbonate and formation of the desired product. The reaction mixture was filtered to remove excess Gabapentin and then filtrate added to ether and cooled at 2° C to afford crystallization of the product. The product was purified by washing with 3 x 25 mL diethyl ether and removing the top ether layer from the solid after each wash. Compounds were subsequently purified by reverse-phase chromatography (30-100% ACN in H_2O). Both products were dried under a flow of N_2 to afford **19-20** as blue solids (36-47%).

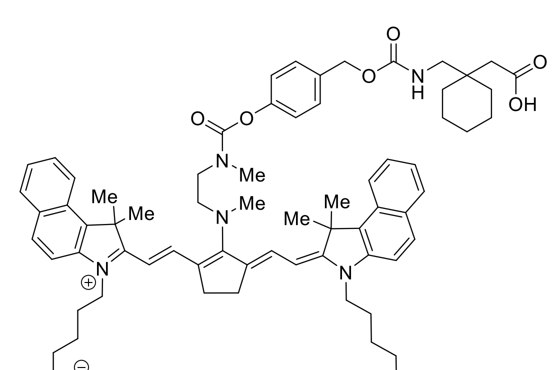

19

19: Synthesized with **17** according to the general procedure outline above to afford solid blue product (0.035 g, 36%). **LC-MS Analysis**: λ_{max}=715 nm, MS: [M+H] 1212, [(MW+2)/2)] 606; [M-H] 1210 (**MW**: 1234.5 g/mol). **:** **^{1}H NMR** (600 MHz, Methanol-d_4): δ 8.08 (d, *J* = 7.2 Hz, 1H), 8.03 (d, *J* = 8.5 Hz, 1H), 7.94-7.82 (m, 6H), 7.57-7.51 (m, 3H), 7.48-7.42 (m, 3H), 7.35 (t, *J* = 7.4 Hz, 3H), 6.94 (d, *J* = 6.7 Hz, 1H), 5.74 (d, *J* = 12.0 Hz, 2H), 4.29 (s, 1H), 4.15-4.03 (m, 4H), 3.79 (s, 1H), 3.75-3.64 (m, 2H), 3.22 (s, 4H), 3.05-2.95 (m, 2H), 2.95-2.78 (m, 10H), 2.03-1.88 (m, 12H), 1.86-1.73 (m, 5H), 1.66 (s, 7H), 1.52-1.26 (m, 10H).

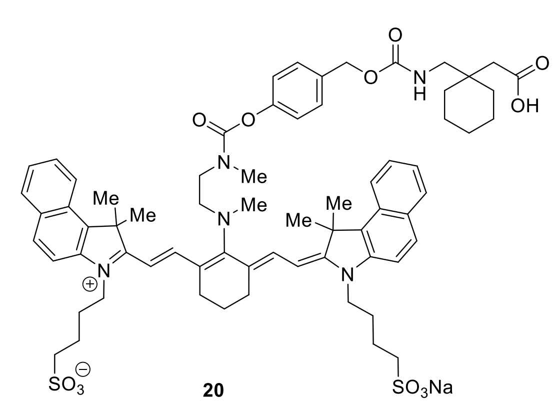

20

20: Synthesized with **18** according to the general procedure outline above to afford solid blue product (0.052 g, 47%). **LC-MS Analysis**: λ_{max}=733 nm, MS: [M-H] 1224 (**MW**: 1248.5 g/mol).

1.5 Synthesis of Hydroxy-Coumarin 26

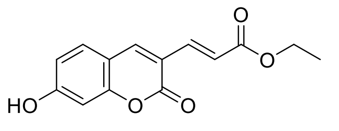

21

21: To a solution of 2,4-dihydroxybenzaldehyde (1.413g, 10.3mmol) in ethanol (30mL) in an oven-dried 100mL round bottom flask was added diethylglutaconate (1.9mL, 10.7mmol) and catalytic piperidine (3 drops). The reaction mixture was refluxed under Argon atmosphere for 24 hours. The now red solution was cooled to room temperature and then cooled further to -20°C for one hour. The resulting yellow precipitate was isolated by vacuum filtration and dried to yield a powdery yellow solid (2.1342g, 80%). **^{1}H NMR** (400 MHz, DMSO-d_6) δ 10.90 (s, 1H), 8.42 (s, 1H), 7.60 – 7.44 (m, 2H), 6.88 – 6.79 (m, 2H), 6.73 (dd, J = 2.2, 0.6 Hz, 1H), 4.16 (q, J = 7.1 Hz, 2H), 1.24 (t, J = 7.1 Hz, 3H). **^{13}C NMR** (101 MHz, DMSO-d_6) δ 166.70, 163.31, 159.66, 155.81, 145.95, 131.35, 120.19, 116.73, 114.48, 111.98, 102.41, 102.39, 60.52, 14.64.

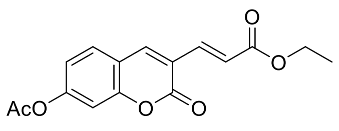

22

22: Phenol **21** (2.1178g, 8.2mmol) was dissolved in pyridine (15mL) in a 100mL round bottom flask and then charged dropwise with acetic anhydride (15mL) and stirred at room temperature for 30 minutes. The reaction mixture was then poured into an ice water mixture and stirred for 10 minutes. The resulting precipitate was collected by vacuum filtration and dried overnight in a vacuum oven to yield a white solid (2.42g, 98%). **^{1}H NMR** (400 MHz, DMSO-d_6) δ 8.56 (s, 1H), 7.77 (d, J = 8.5 Hz, 1H), 7.54 (dd, J = 16.0, 0.6 Hz, 1H), 7.35 – 7.30 (m, 1H), 7.20 (dd, J = 8.5, 2.2 Hz, 1H), 6.97 – 6.89 (m, 1H), 4.19 (q, J = 7.1 Hz, 2H), 2.30 (s, 3H), 1.25 (t, J = 7.1 Hz, 3H). **^{13}C NMR** (101 MHz, DMSO-d_6) δ 169.15, 166.41, 159.13, 154.28, 154.15, 144.67, 130.62, 122.23, 120.89, 117.27, 110.39, 109.99, 60.73, 25.24, 21.32, 14.58.

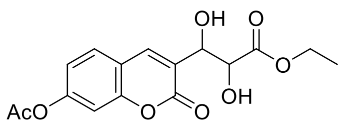

23

23: α,β-unsaturated ester **22** (0.930g, 3.08mmol) was dissolved in dichloromethane (15mL) and charged with a 1:1 w/w solution of N-methylmorpholine-N-oxide (0.721g, 6.16mmol) in water (0.8mL) and 3.0mL of 2.5wt% OsO_4 in t-BuOH. The solution was stirred at room temperature for 10 hours until completion was confirmed by TLC. The reaction was then quenched with water (50mL) and extracted with ethyl acetate (3 x 30mL). The combined organic layers were washed with brine (30mL), dried over Na_2SO_4, filtered, and concentrated under reduced pressure to yield a white solid (0.7486g, 72%). **^{1}H NMR** (400 MHz, DMSO-d_6) δ 8.02 (s, 1H), 7.83 (d, J = 8.5 Hz, 1H), 7.30 (d, 1H), 7.17 (dd, J = 8.4, 2.2 Hz, 1H), 5.72 (d, J = 6.7 Hz, 1H), 5.18 (d, J = 7.1 Hz, 1H), 4.99 – 4.93 (m, 1H), 4.39 (dd, J = 7.0, 2.6 Hz, 1H), 4.03 (q, J = 7.1 Hz, 2H), 2.31 (s, 3H), 1.17 (t, J = 7.1 Hz, 3H). **^{13}C NMR** (101 MHz, DMSO-d_6) δ 171.78, 168.84, 159.53, 153.05, 152.38, 139.36, 129.14, 128.00, 116.93, 110.31, 109.81, 71.36, 60.30, 20.91, 20.75, 14.18.

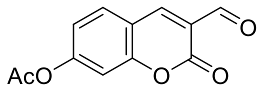

24

24: Diol **23** (0.7486g 2.23mmol) was added to a slurry of silica gel (5g) in dichloromethane (10mL). While stirring vigorously, the reaction mixture was charged dropwise with a solution of sodium periodate ($NaIO_4$, 0.6333g, 2.96mmol) over 5 minutes and then stirred at room temperature for 2 hours. The reaction mixture was filtered through a cake of celite and washed with copious amounts of ethyl acetate. The ethyl acetate was washed with water (30mL) and brine (30mL), dried over Na_2SO_4, filtered, and dry-loaded onto celite. The crude reaction mixture was purified by silica gel chromatography using a 5-50% ethyl acetate in hexane, and the product eluted in 45%. The relevant fractions were combined and concentrated under reduced pressure to yield a

white solid (0.396g, 76%). **^{1}H NMR** (400 MHz, DMSO-d_6) δ 10.03 (s, 1H), 8.71 – 8.68 (m, 1H), 8.07 – 8.01 (m, 1H), 7.41 – 7.37 (m, 1H), 7.27 (dd, *J* = 8.5, 2.2 Hz, 1H), 2.33 (s, 3H).

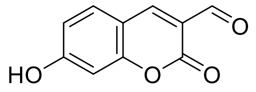

25

25: Aldehyde **24** (0.395g, 1.7mmol) was dissolved in 30% w/w NH_4OH in water (5mL) and charged with acetonitrile (5mL) slowly. The solution was then stirred 10 minutes at room temperature before being quenched with ethyl acetate (30mL). The organic layer was then washed with 1M HCl (2 x 20mL), dried over Na_2SO_4, filtered, and concentrated under reduced pressure to yield a yellow solid (0.2605g, 81%). **^{1}H NMR** (400 MHz, DMSO-d_6) δ 9.96 (s, 1H), 8.61 – 8.56 (m, 1H), 7.82 (d, *J* = 8.6 Hz, 1H), 6.88 (dd, *J* = 8.6, 2.3 Hz, 1H), 6.80 – 6.76 (m, 1H).

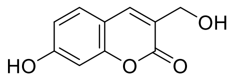

26

26: Phenol **25** (0.0541g, 0.28mmol) was dissolved in methanol (5mL) and cooled to 0°C. Solid $NaBH_4$ (0.013g, 0.34mmol) was added slowly, and the reaction mixture was stirred at 0°C for 30 minutes. The mixture was then diluted with ethyl acetate (20mL) and washed with saturated NH_4Cl(aq) solution (20mL), dried over Na2SO4, filtered, and concentrated under reduced pressure to yield a yellow solid (0.0465g, 85%).

2 NMR and LC-MS Spectra

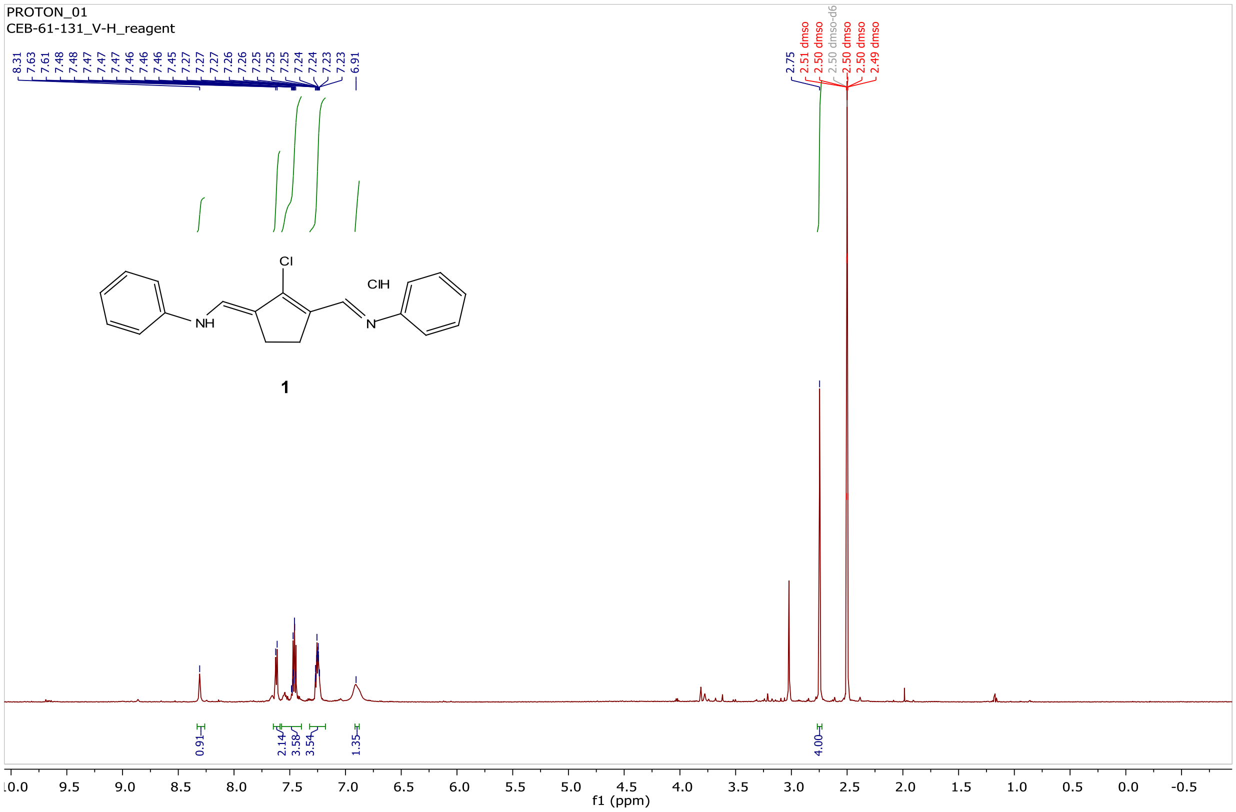

Supplementary Figure 1. ^{1}H NMR spectrum of **1.**

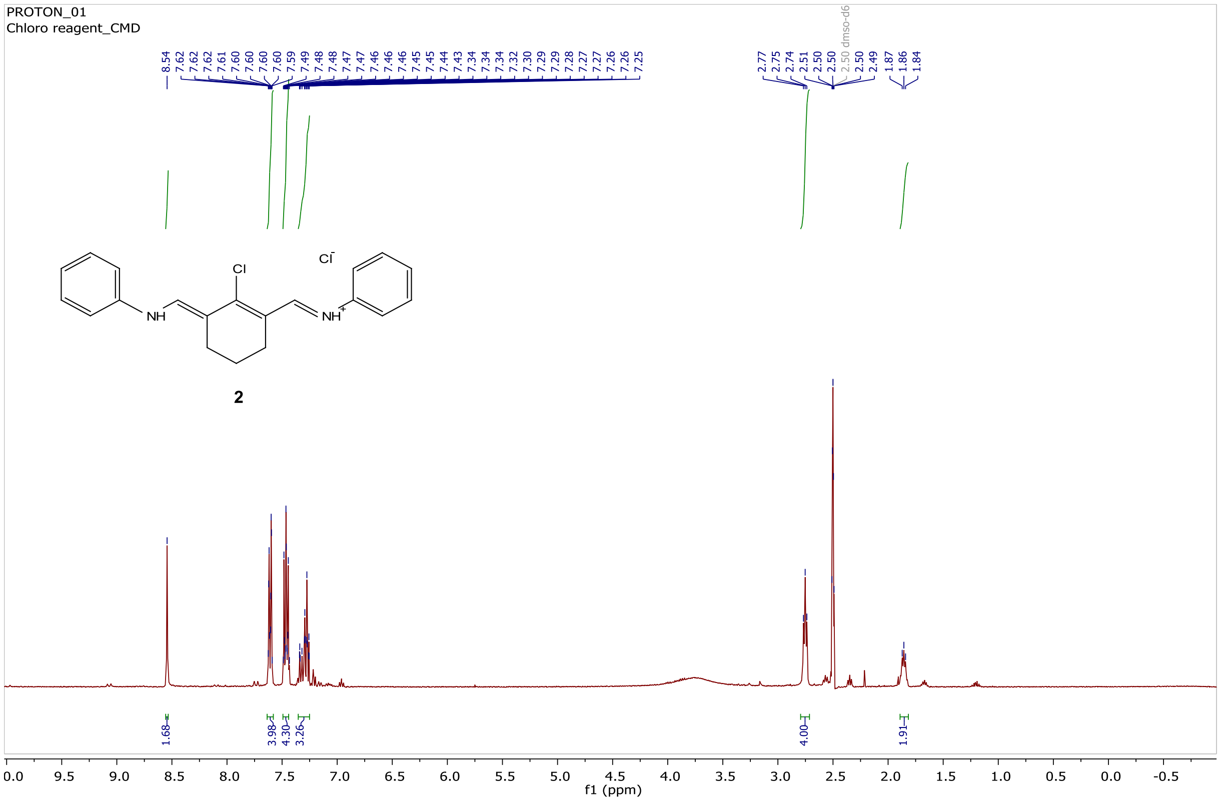

Supplementary Figure 2. ^{1}H NMR spectrum of **2.**

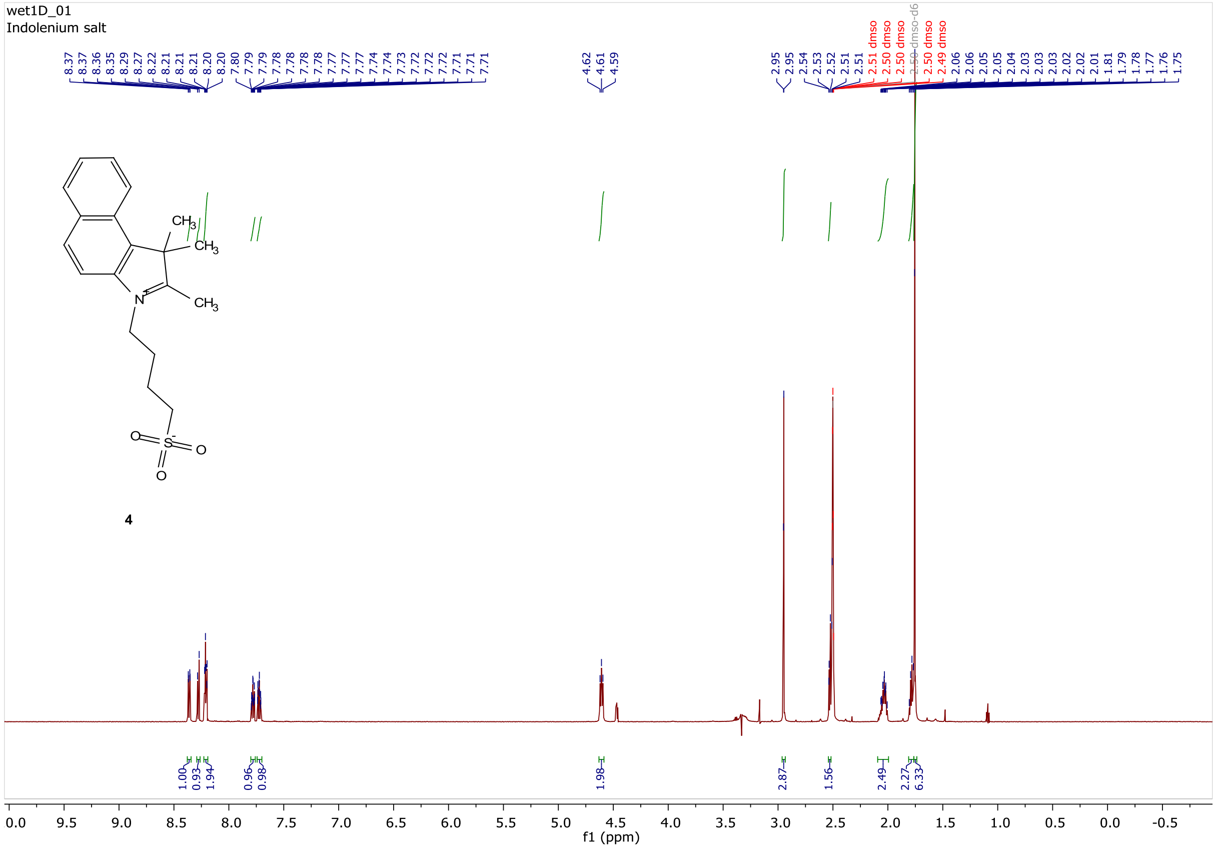

Supplementary Figure 3. ^{1}H NMR spectrum of **4.**

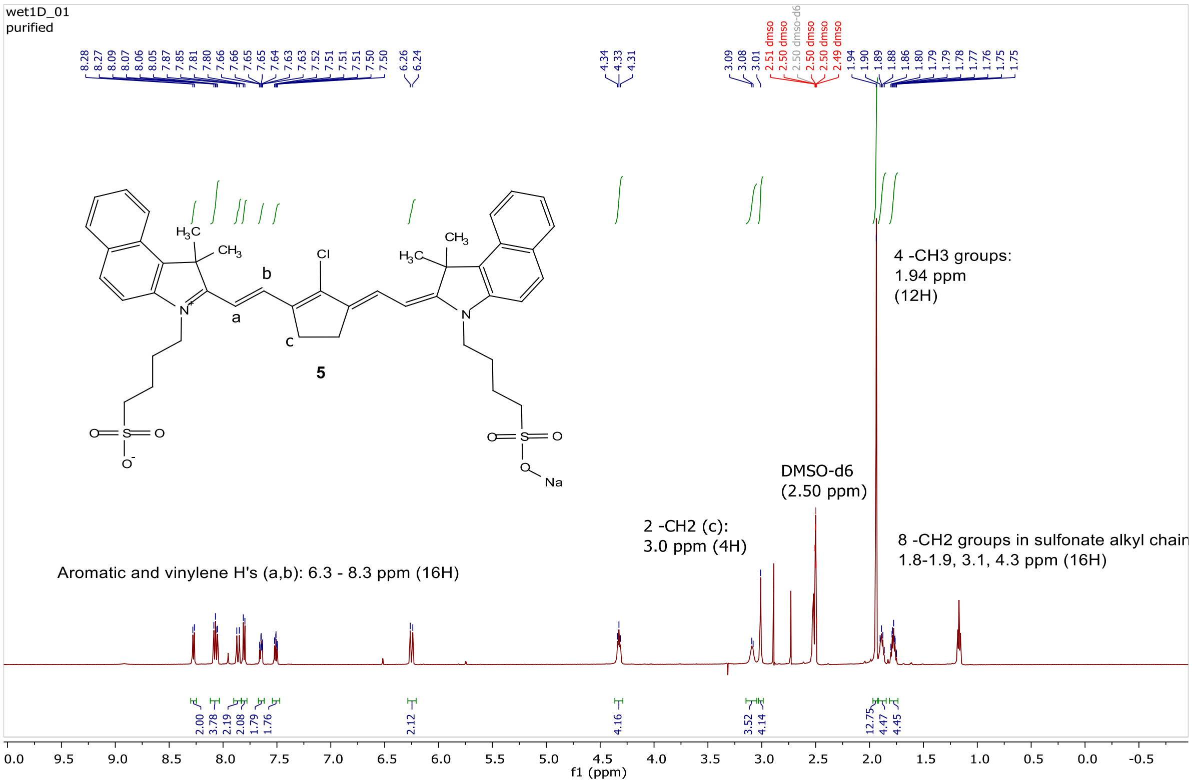

Supplementary Figure 4. ^{1}H NMR spectrum of **5.**

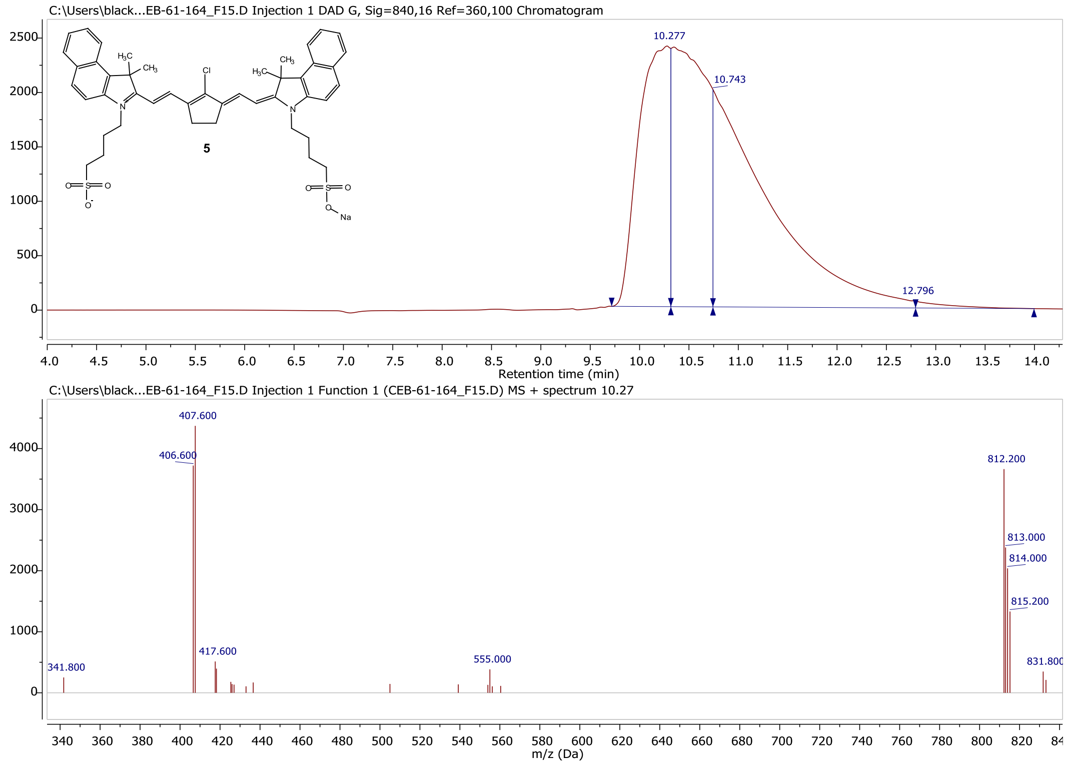

Supplementary Figure 5. LC-MS data of **5**, λ_{max} = 844 nm. *Top:* Chromatogram at 840 nm with pure **5** at T_{ret} = 10.3 minutes. *Bottom:* MS in positive mode with **5** seen at 812.2 [MW without Na^+ ion: 811.26 g/mol].

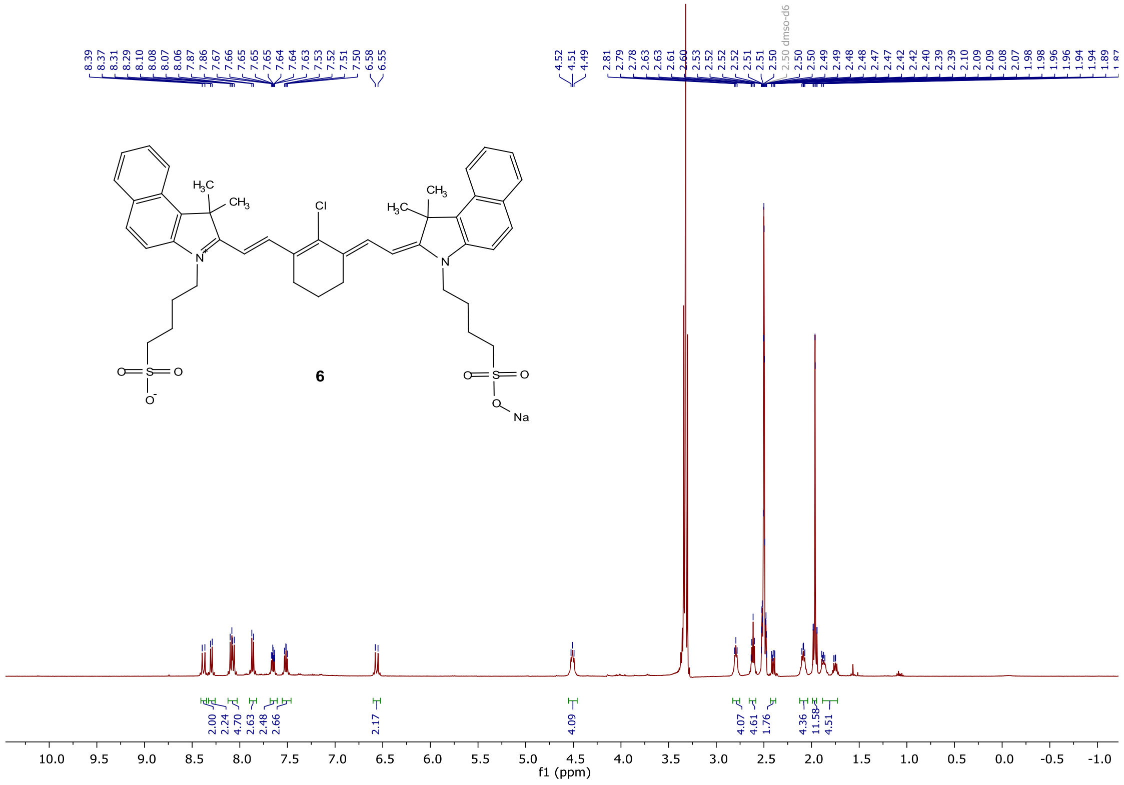

Supplementary Figure 6. ^{1}H NMR spectrum of **6**.

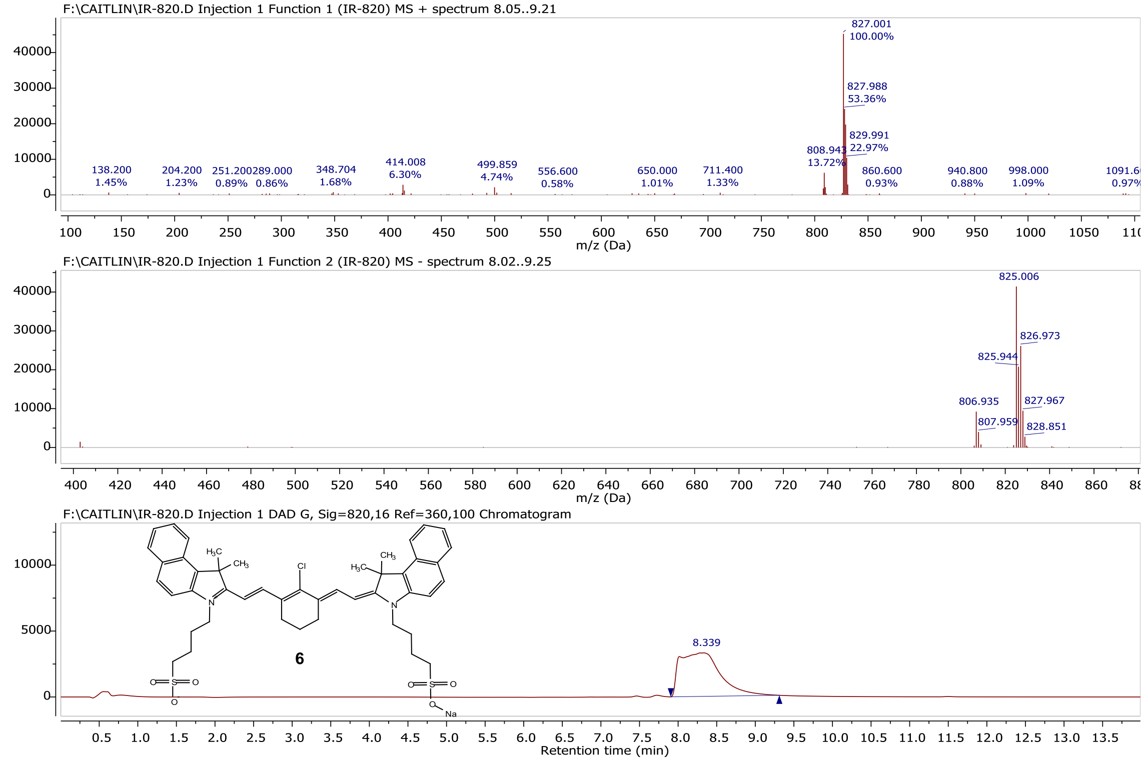

Supplementary Figure 7. LC-MS data of **6**, λ_{max} = 820 nm. *Top:* MS in positive mode with **6** seen at 827.0 [MW without Na^+ ion: 826.5 g/mol]. *Middle:* MS in negative mode with **6** seen at 825.0. *Bottom:* Chromatogram at 820 nm with pure **6** at T_{ret} = 8.0 – 8.9 minutes.

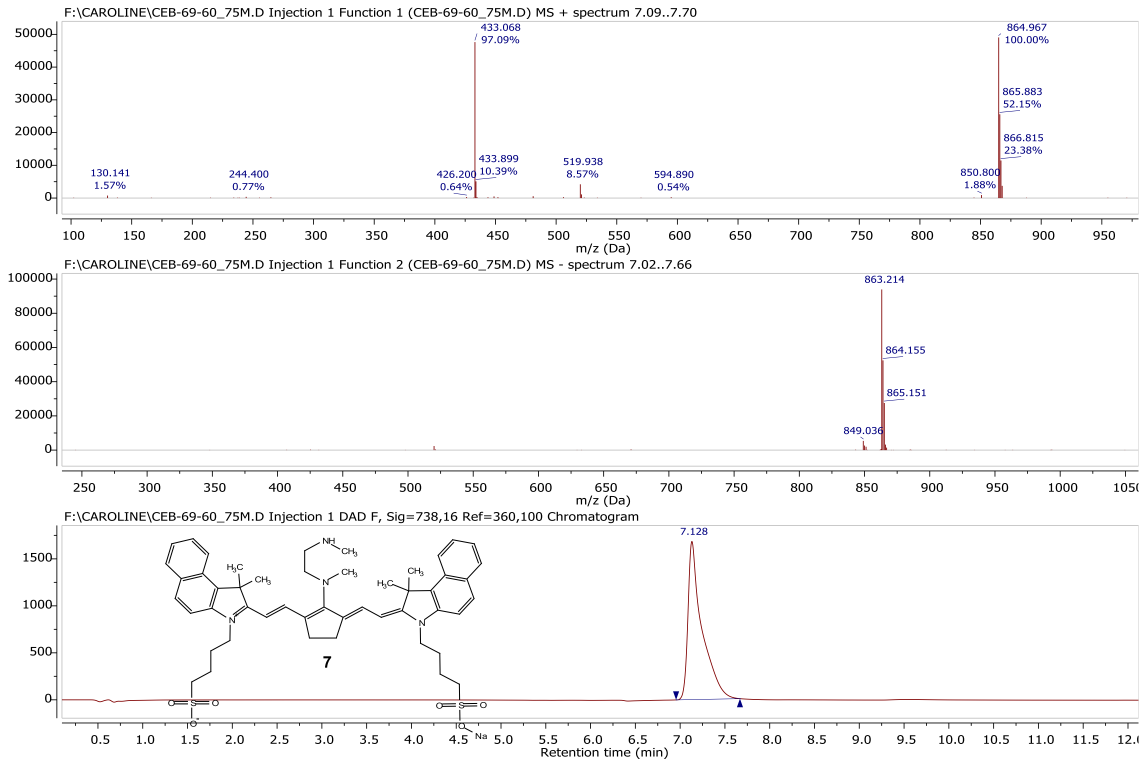

Supplementary Figure 8. LC-MS data of **7**, λ_{max} = 738 nm. *Top*: MS in positive mode with **7** at 865.0 and 433.1 ((MW+2)/2) [MW without Na^+ ion: 864.2 g/mol]. *Middle:* MS in negative mode with **7** at 863.2. *Bottom:* Chromatogram at 738 nm with **7** at T_{ret} = 7.1 minutes.

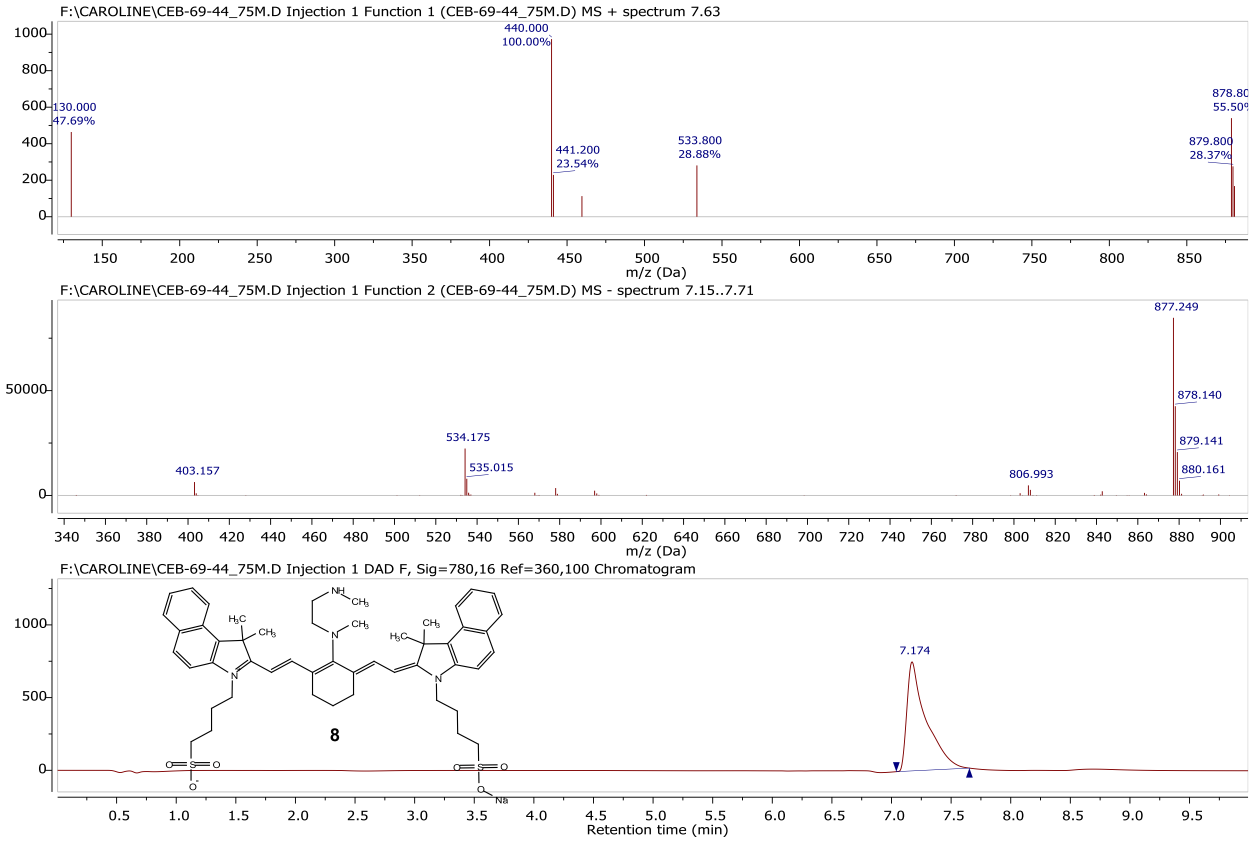

Supplementary Figure 9. LC-MS data of **8**, λ_{max} = 778 nm. *Top*: MS in positive mode with **8** at 878.8 and 440.0 ((MW+2)/2) [MW without Na^+ ion: 878.2 g/mol]. *Middle:* MS in negative mode with **8** at 877.2. *Bottom:* Chromatogram at 780 nm with **8** at T_{ret} = 7.2 minutes.

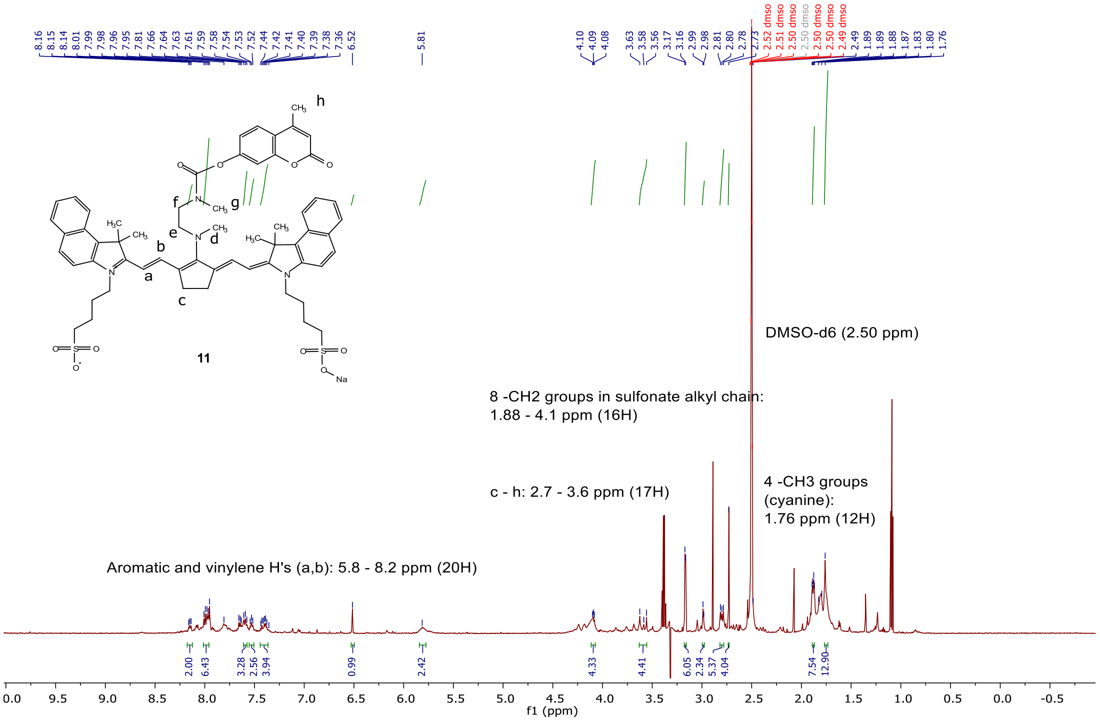

Supplementary Figure 10. ^{1}H NMR spectrum of **11.**

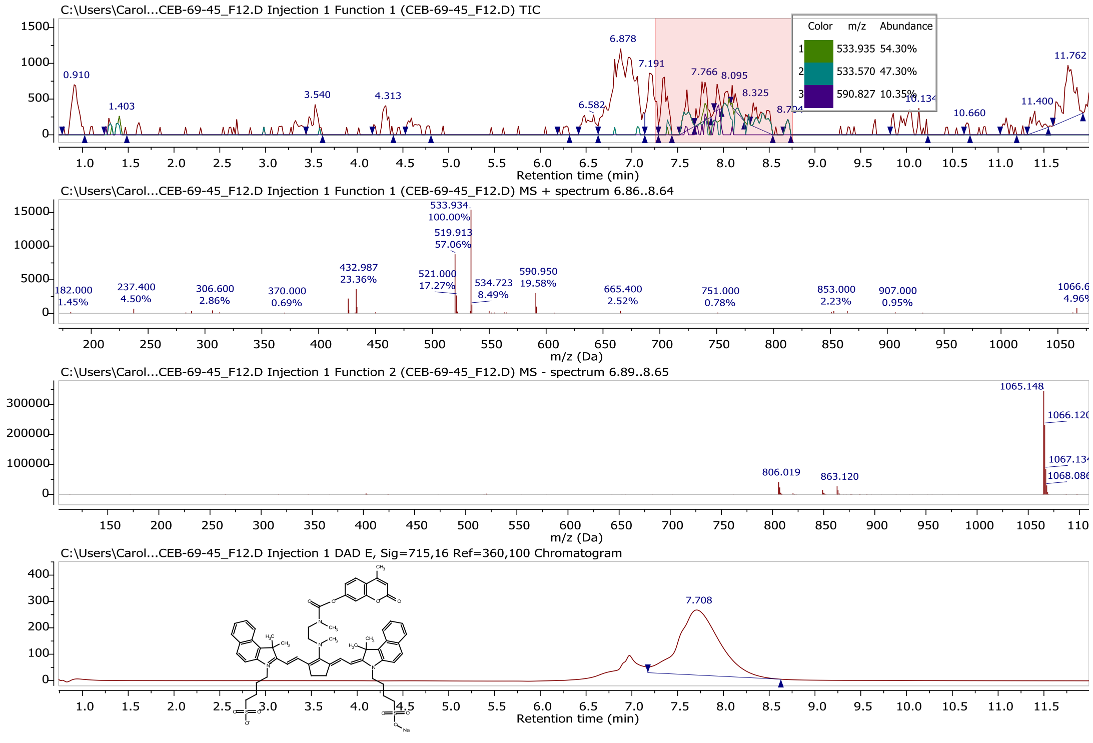

Supplementary Figure 11. LC-MS data of **11,** λ_{max} = 715 nm. *Top:* TIC showing the peak purity of the major LC peak (7.2-8.5 min) to be majority product masses [533.9/533.5 (MW+2)/2]. *Upper middle*: MS in positive mode with **11** at 1066.7 and 533.9 ((MW+2)/2) [MW without Na^+ ion: 1066.3

g/mol]. *Lower middle:* MS in negative mode with **11** at 1065.1. *Bottom:* Chromatogram at 715 nm with **11** at T_{ret} = 7.2-8.5 minutes.

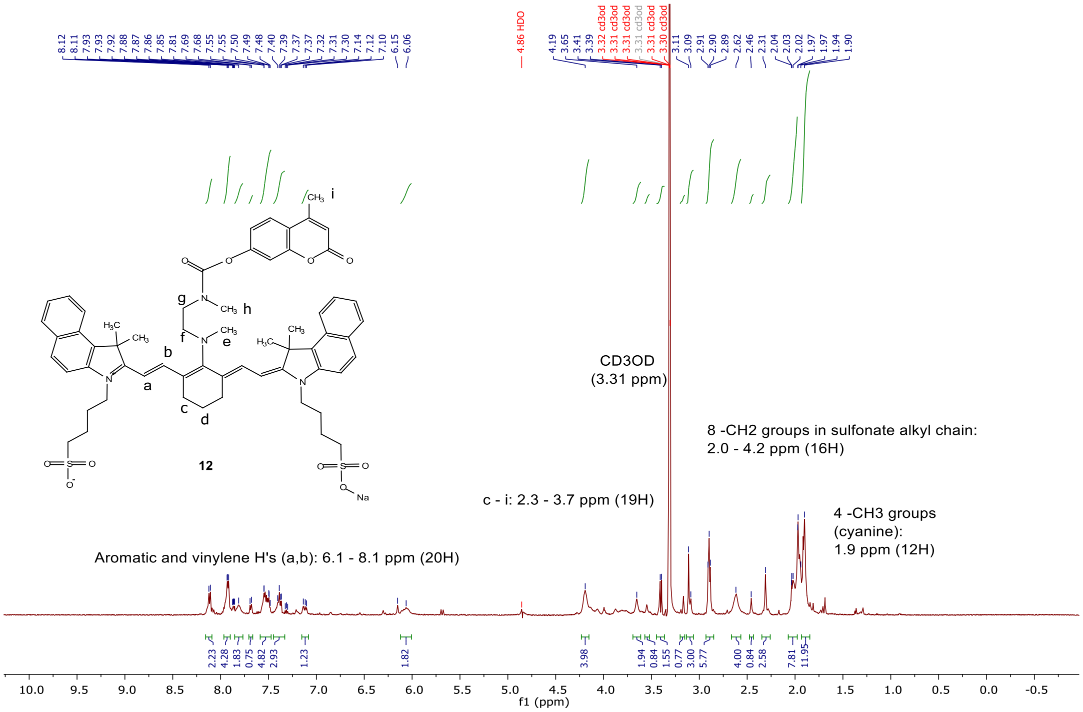

Supplementary Figure 12. ^{1}H NMR spectrum of **12.**

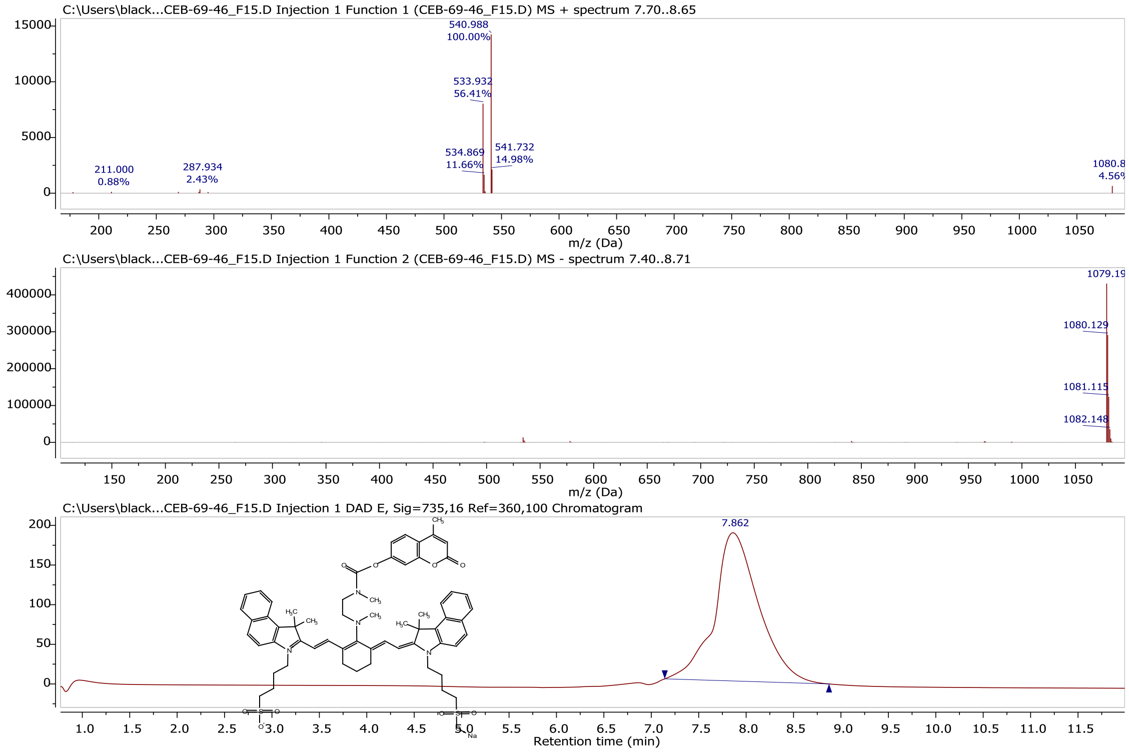

Supplementary Figure 13. LC-MS data of **12,** λ_{max} = 735 nm. *Top:* MS in positive mode with **12** at 1080.8 and 541.0 ((MW+2)/2) [MW without Na^+ ion: 1080.4 g/mol]. *Middle:* MS in negative mode with **12** at 1079.2. *Bottom:* Chromatogram at 735 nm with **12** at T_{ret} = 7.9 minutes.

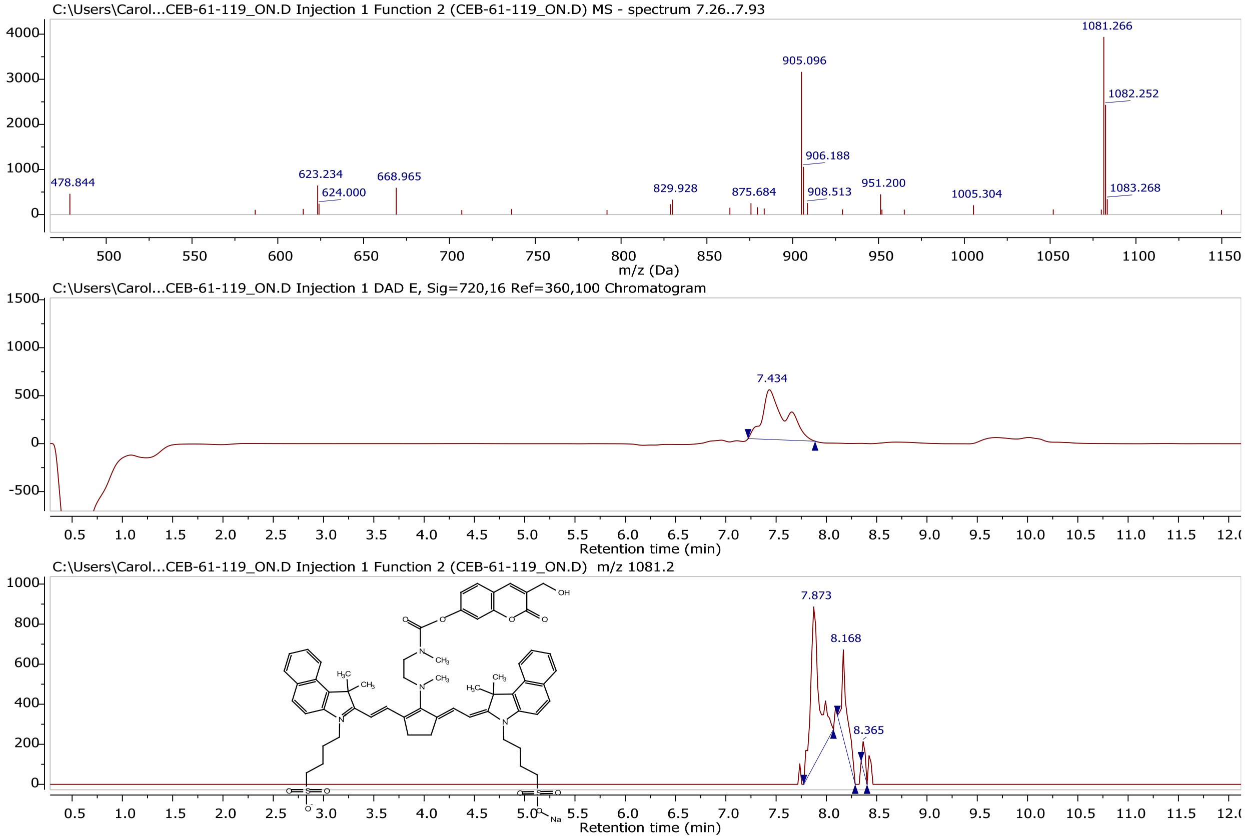

Supplementary Figure 14. LC-MS data of **13**, λ_{max} = 720 nm. *Top:* MS in negative mode with **13** at 1081.3 [MW without Na^+ ion: 1082.3 g/mol]. *Middle:* Chromatogram at 720 nm with **13** at T_{ret} = 7.5 minutes. *Bottom:* EIC of product m/z 1081.2 in MS negative mode.

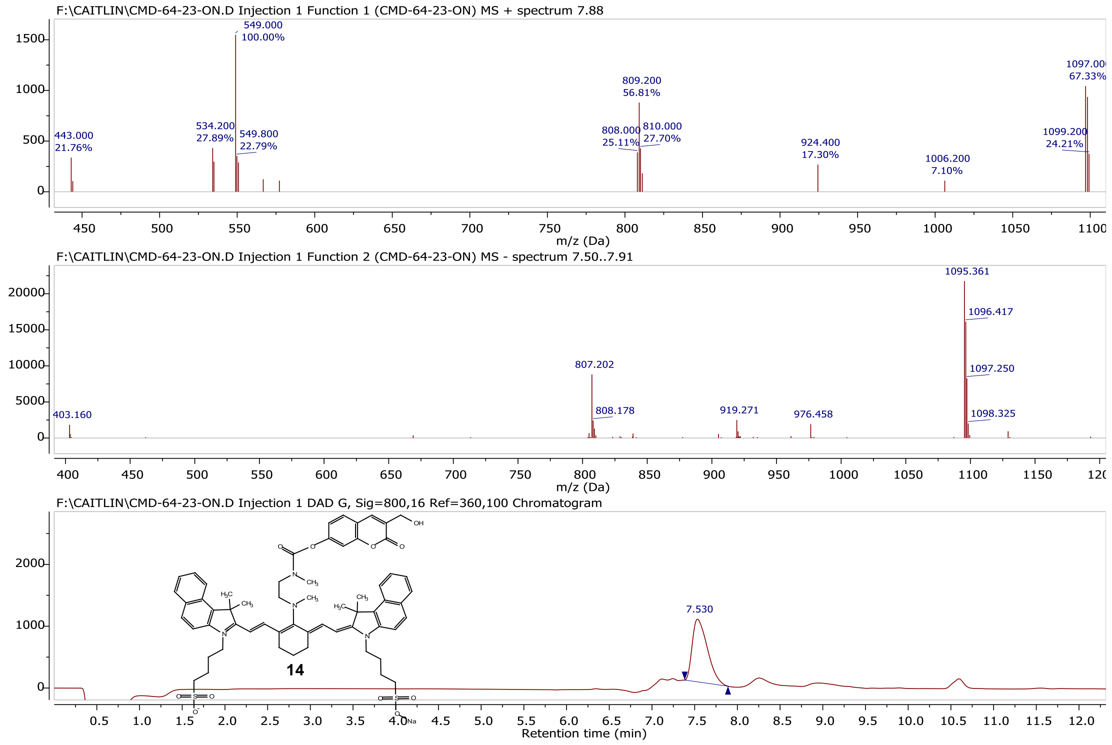

Supplementary Figure 15. LC-MS data of **14**, λ_{max} = 743 nm. *Top:* MS in positive mode with **14** at 1097.0 and 549.0 ((MW+2)/2) [MW without Na^+ ion: 1096.3 g/mol]. *Middle:* MS in negative mode with **14** at 1095.4. *Bottom:* Chromatogram at 800 nm with **14** at T_{ret} = 7.5 minutes.

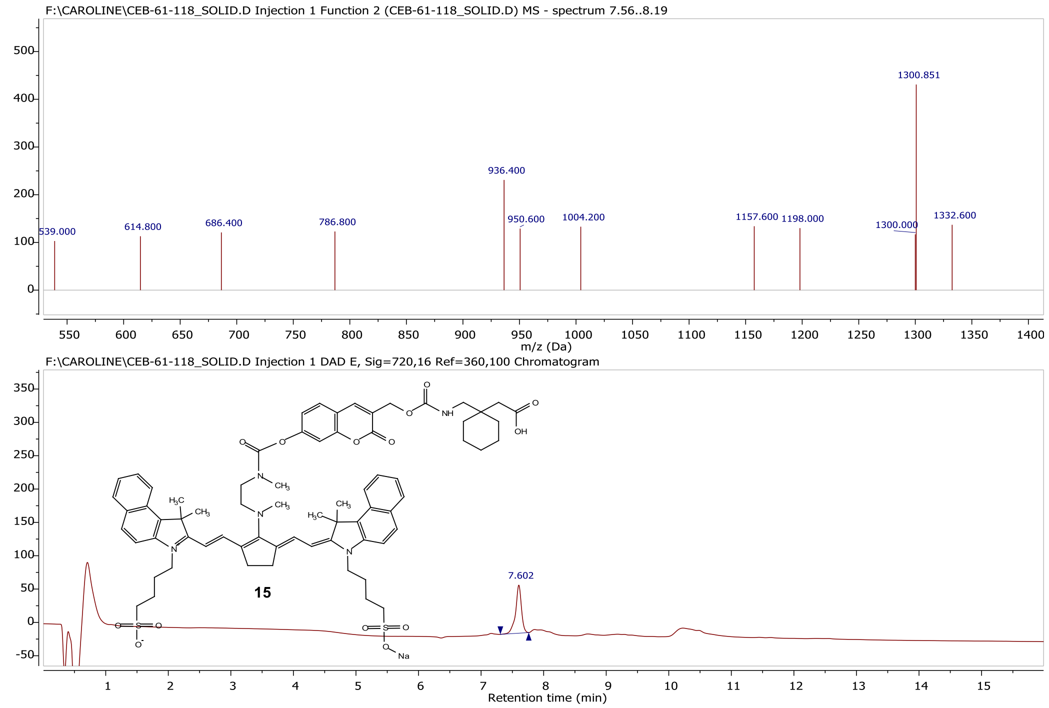

Supplementary Figure 16. LC-MS data of **15,** λ_{max} = 715 nm. *Top:* MS in negative mode with **15** at 1300.9 [MW: 1301.5 g/mol]. *Bottom*: Chromatogram at 720 nm with **15** at T_{ret} = 7.6 – 8 minutes.

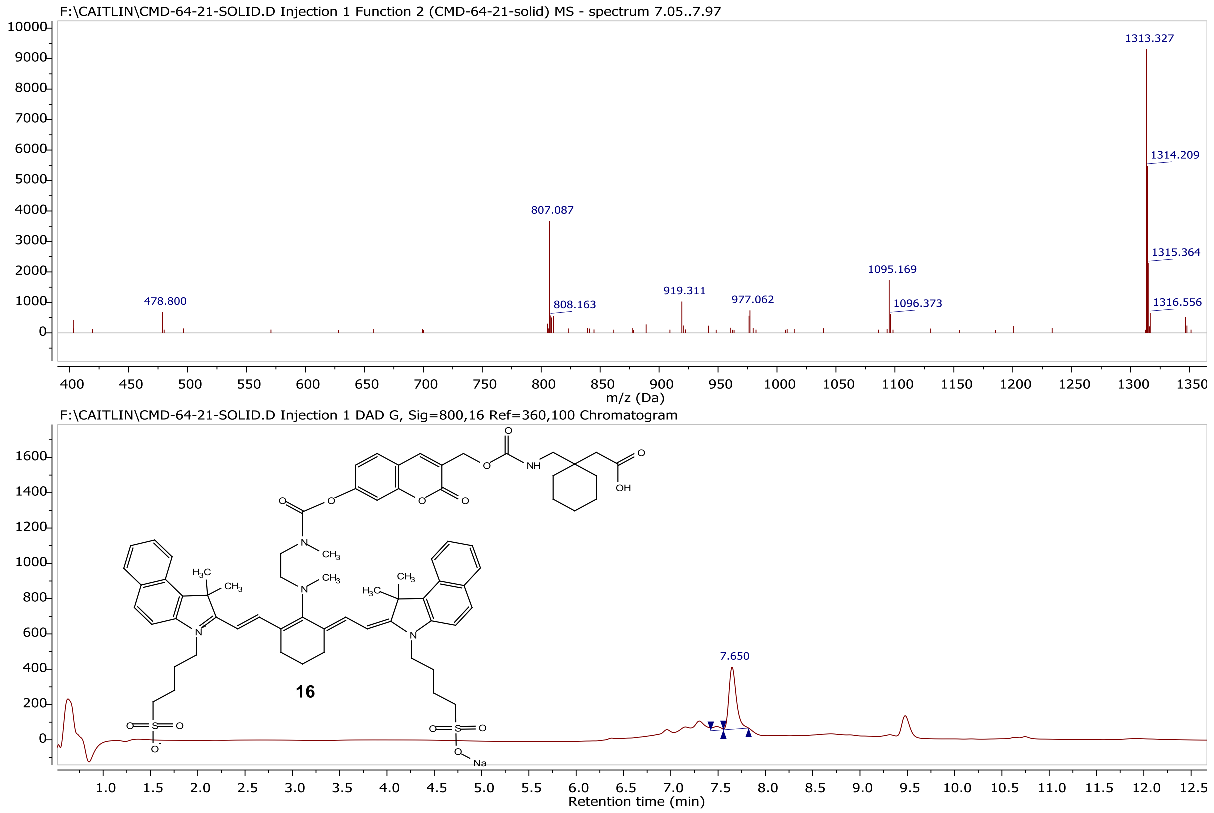

Supplementary Figure 17. LC-MS data of **16**, λ_{max} = 735 nm. *Top:* MS in negative mode with **16** at 1313.3/1314.2 [MW: 1315.5 g/mol]. *Bottom*: Chromatogram at 800 nm with **16** at T_{ret} = 7. – 8 minutes.

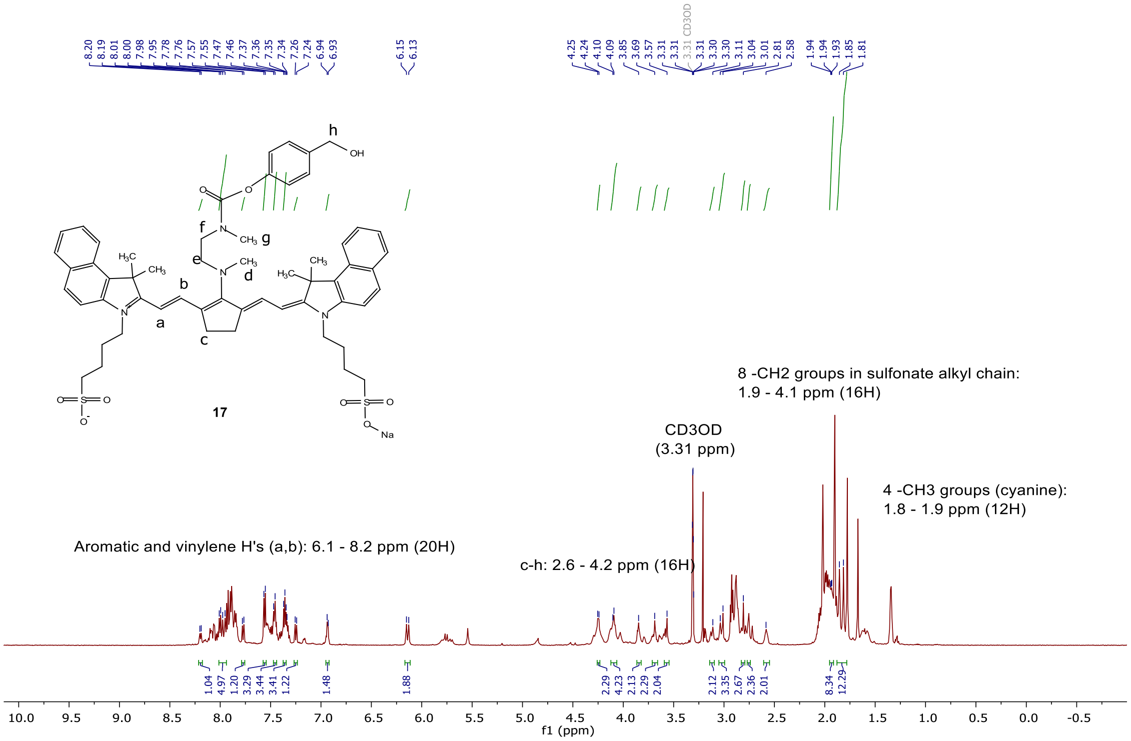

Supplementary Figure 18. ^{1}H NMR spectrum of **17.**

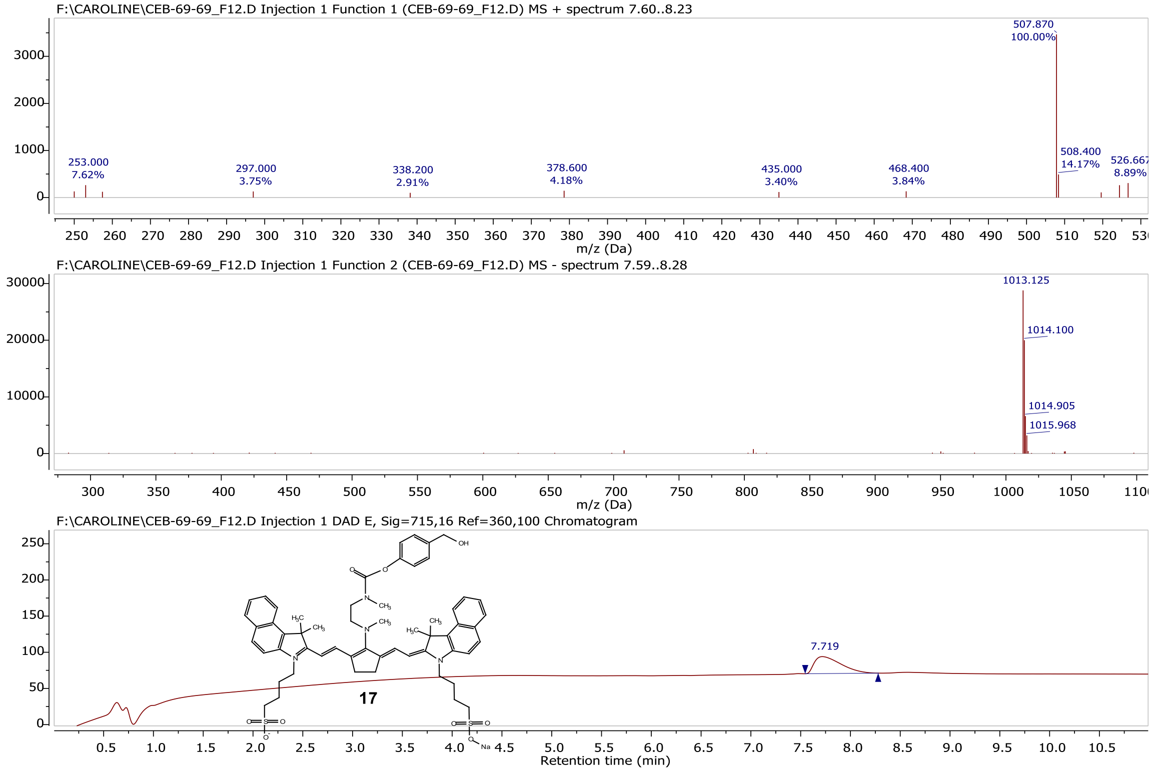

Supplementary Figure 19. LC-MS data of **17,** λ_{max} = 718 nm. *Top*: MS in positive mode with **17** at 507.9 ((MW+2)/2) [MW without Na^+ ion: 1014.3 g/mol]. *Middle:* MS in negative mode with **17** at 1013.1. *Bottom:* Chromatogram at 715 nm with **17** at T_{ret} = 7.7 minutes.

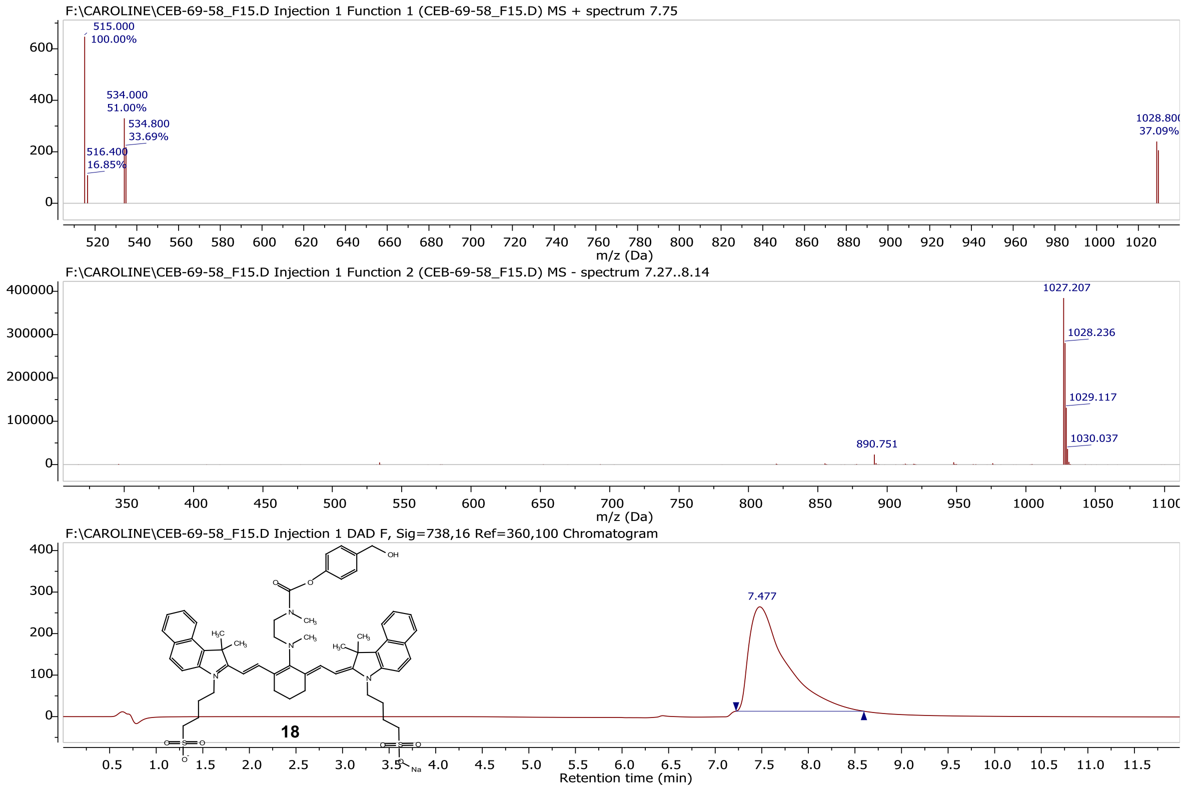

Supplementary Figure 20. LC-MS data of **18**, λ_{max} = 738 nm. *Top*: MS in positive mode with **18** at 1028.8 and 515.0 ((MW+2)/2) [MW without Na^+ ion: 1028.3 g/mol]. *Middle:* MS in negative mode with **18** at 1027.2. *Bottom:* Chromatogram at 738 nm with **18** at T_{ret} = 7.5 minutes.

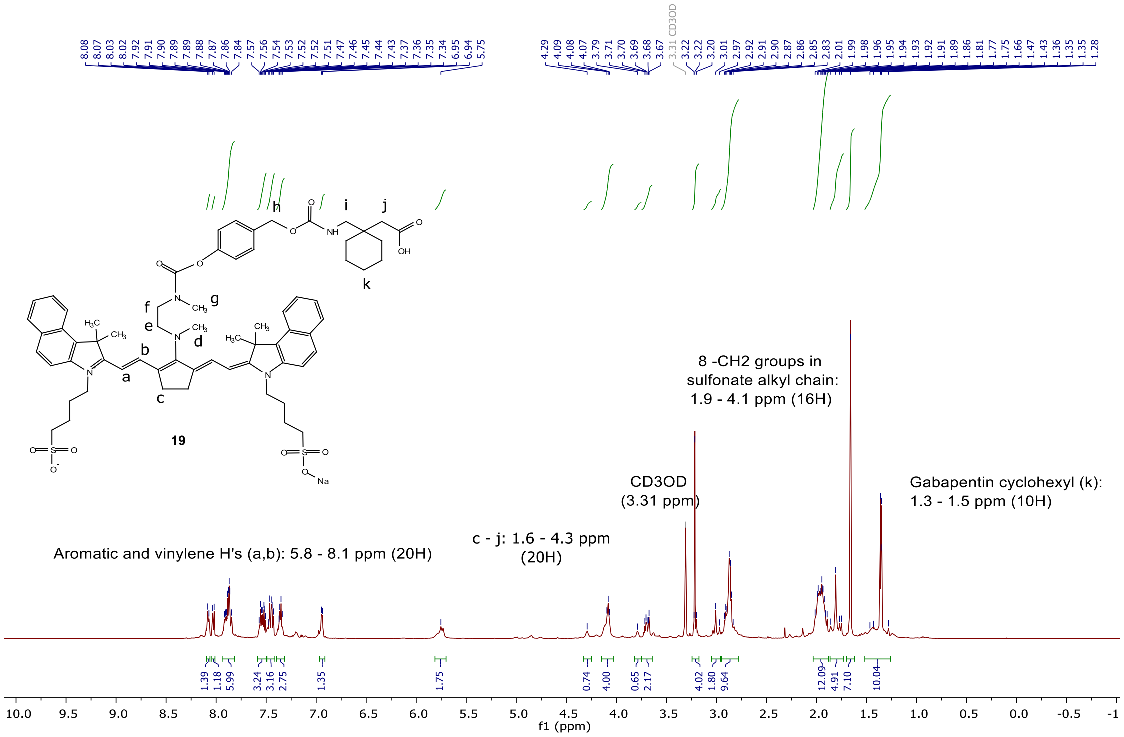

Supplementary Figure 21. ^{1}H NMR spectrum of **19.**

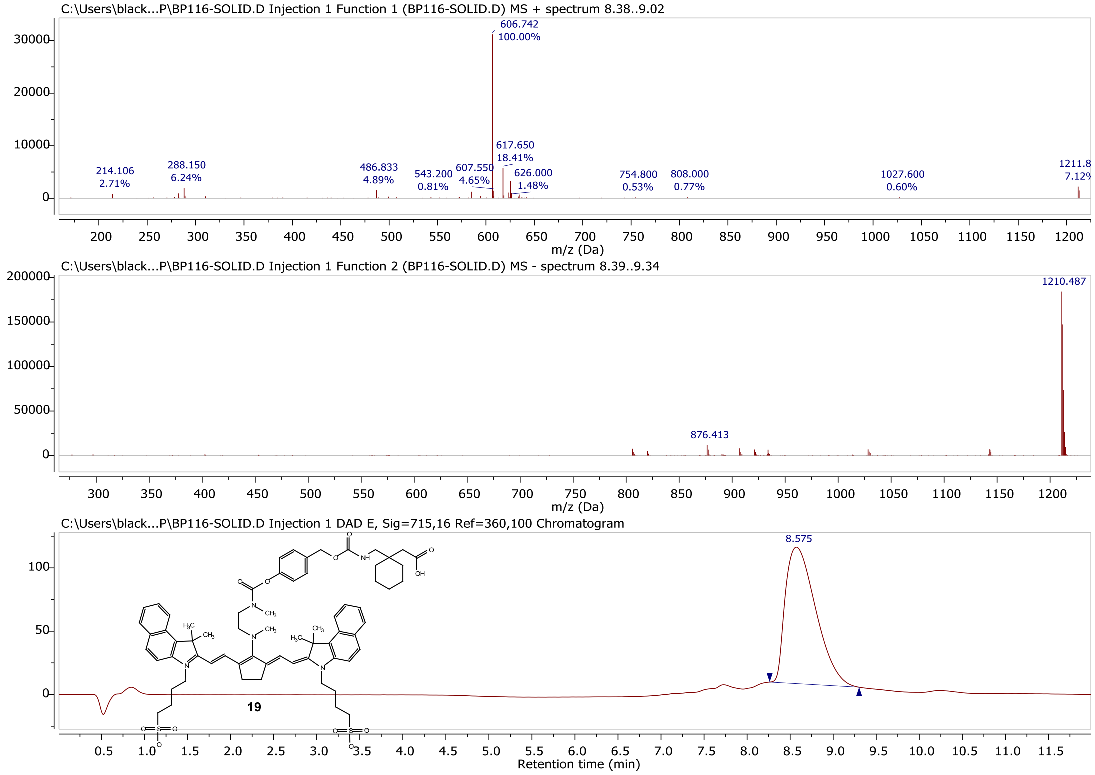

Supplementary Figure 22. LC-MS data of **19,** λ_{max} = 715 nm. *Top:* MS in positive mode with **19** at 1211.9 and 606.7 ((MW+2)/2) [MW without Na^{+} ion: 1211.5 g/mol]. *Middle:* MS in negative mode with **19** at 1210.5. *Bottom:* Chromatogram at 715 nm with **19** at T_{ret} = 8.6 minutes.

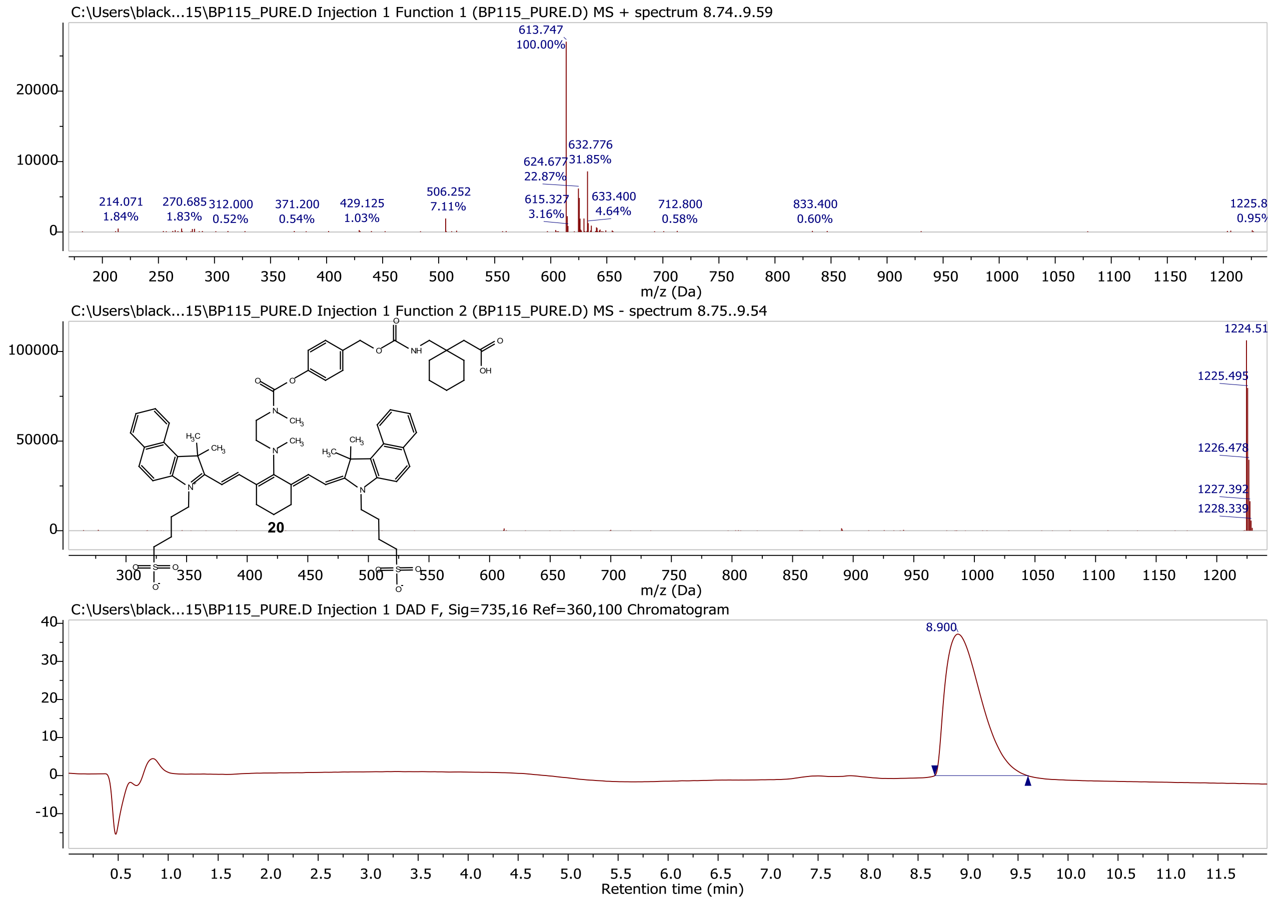

Supplementary Figure 23. LC-MS data of **20,** λ_{max} = 733 nm. Top: MS in positive mode with **20** at 1225.8 and 613.7 ((MW+2)/2) [MW without Na^+ ion: 1225.6 g/mol]. Middle: MS in negative mode with **20** at 1224.5. Bottom: Chromatogram at 735 nm with **20** at T_{ret} = 8.9 minutes.

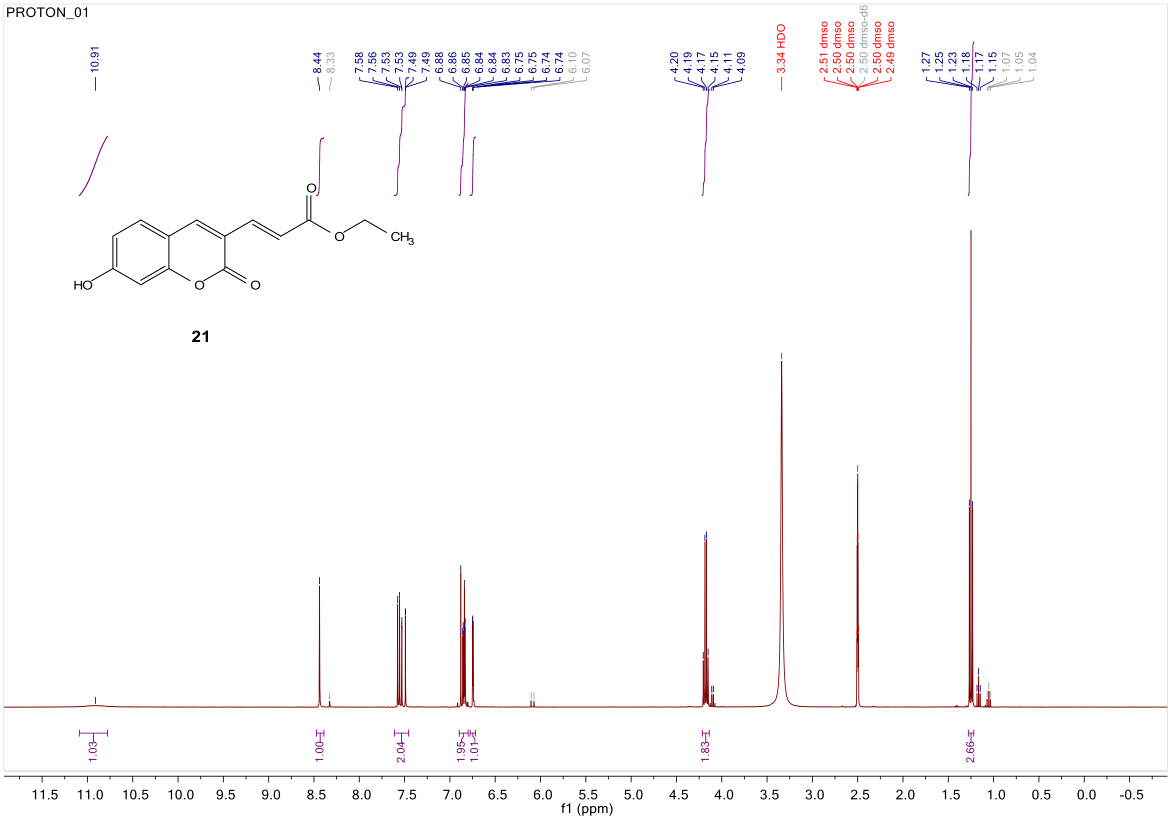

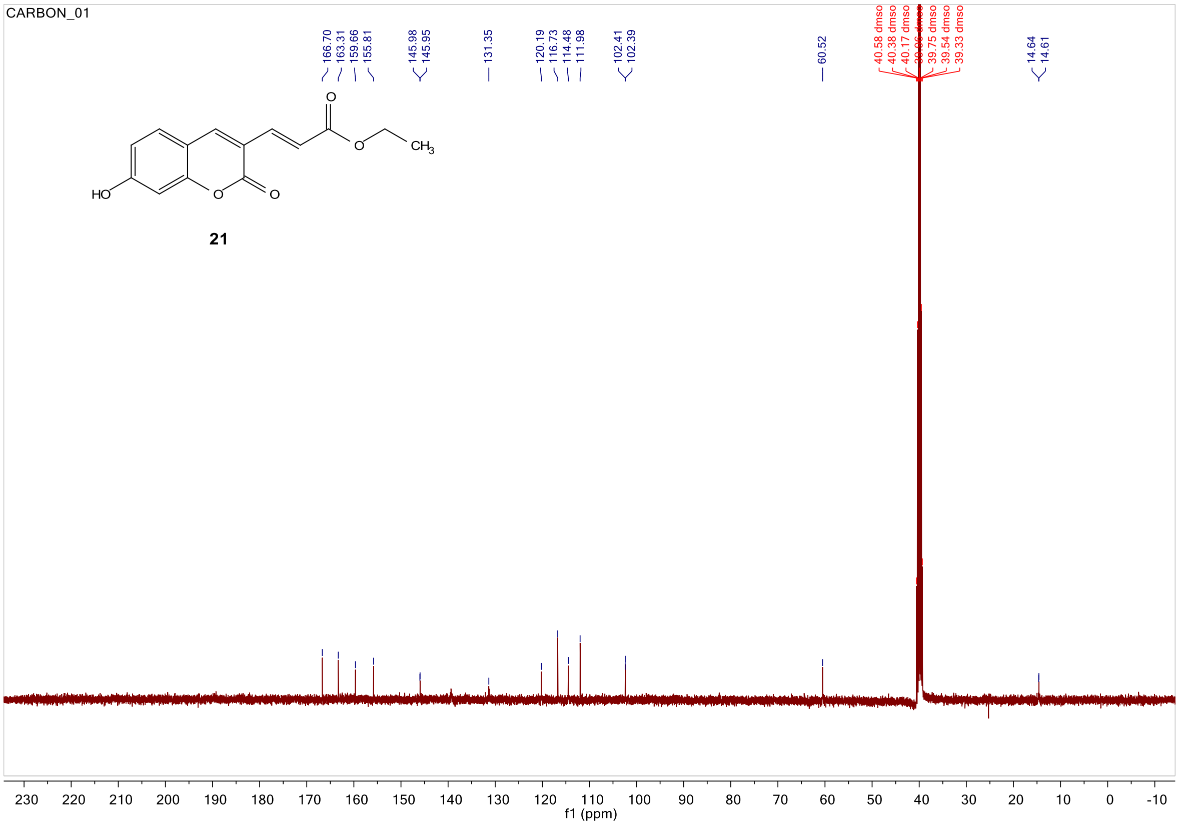

Supplementary Figure 24. ^{1}H and ^{13}C NMR spectra of **21.**

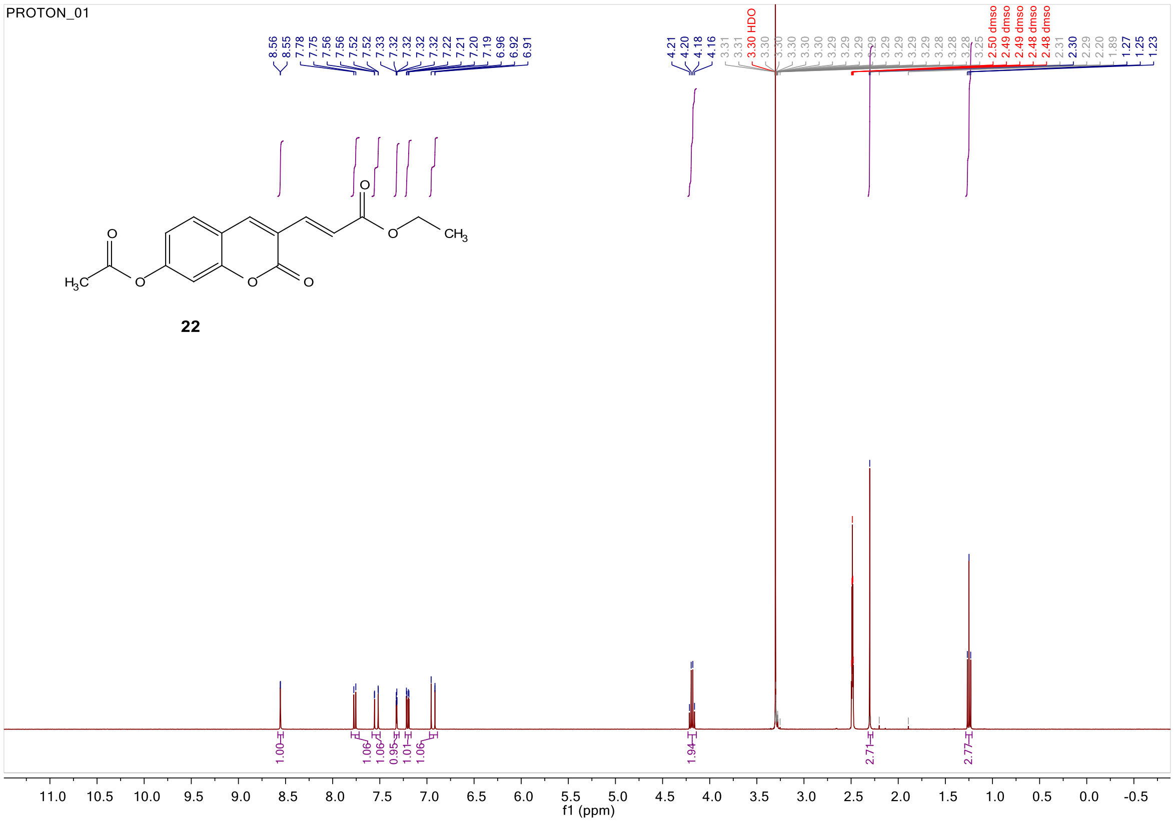

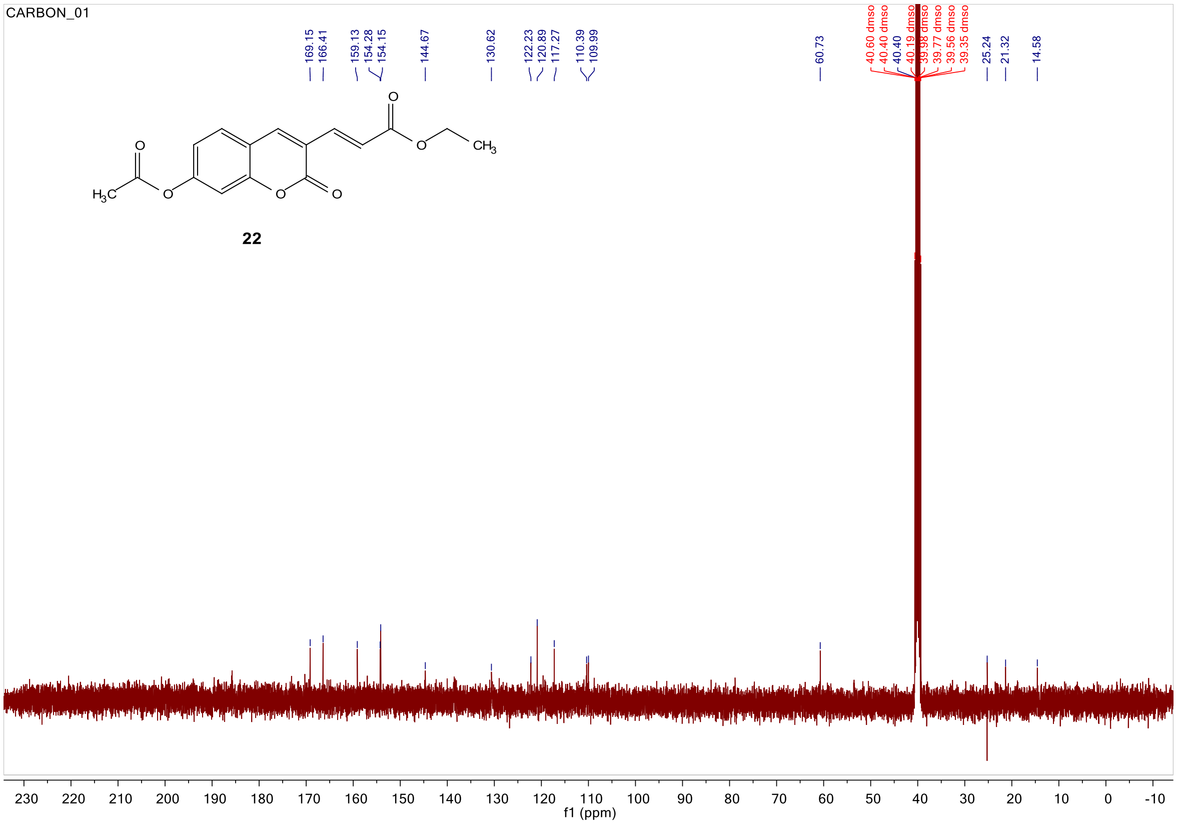

Supplementary Figure 25. ^{1}H and ^{13}C NMR spectra of **22.**

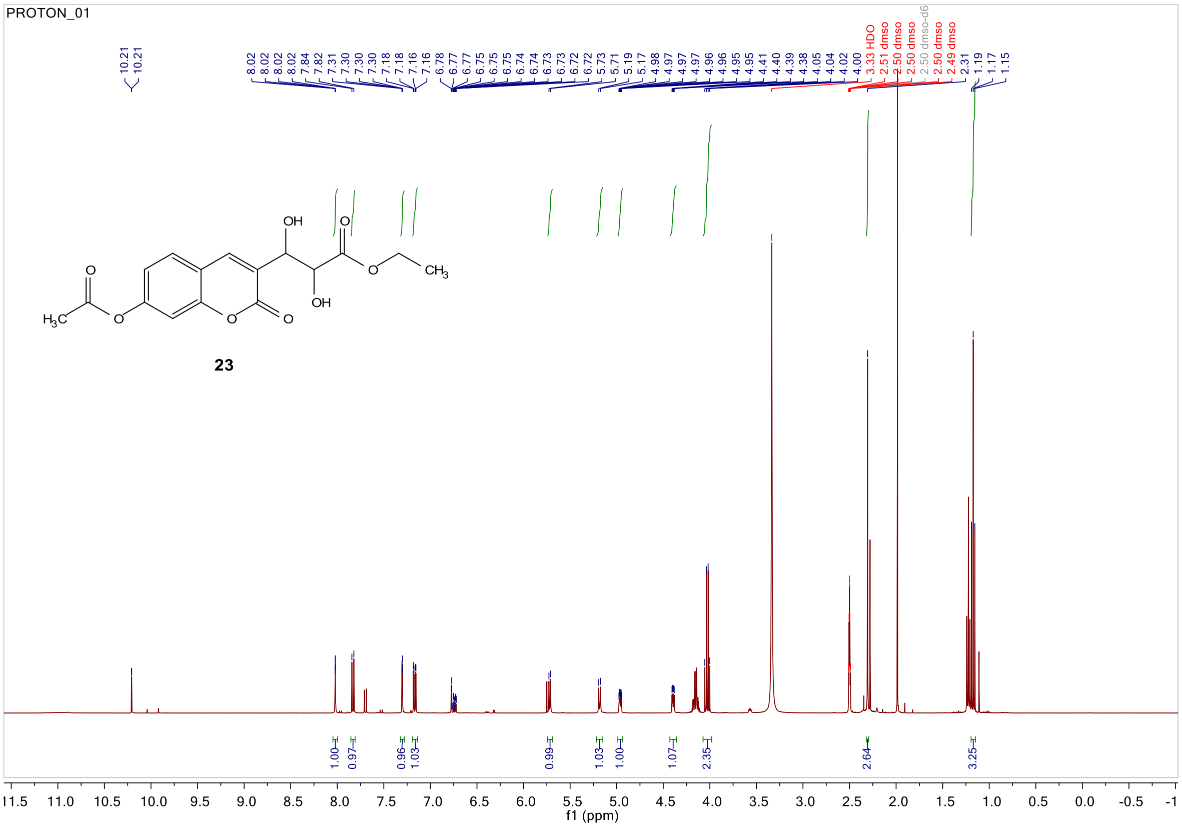

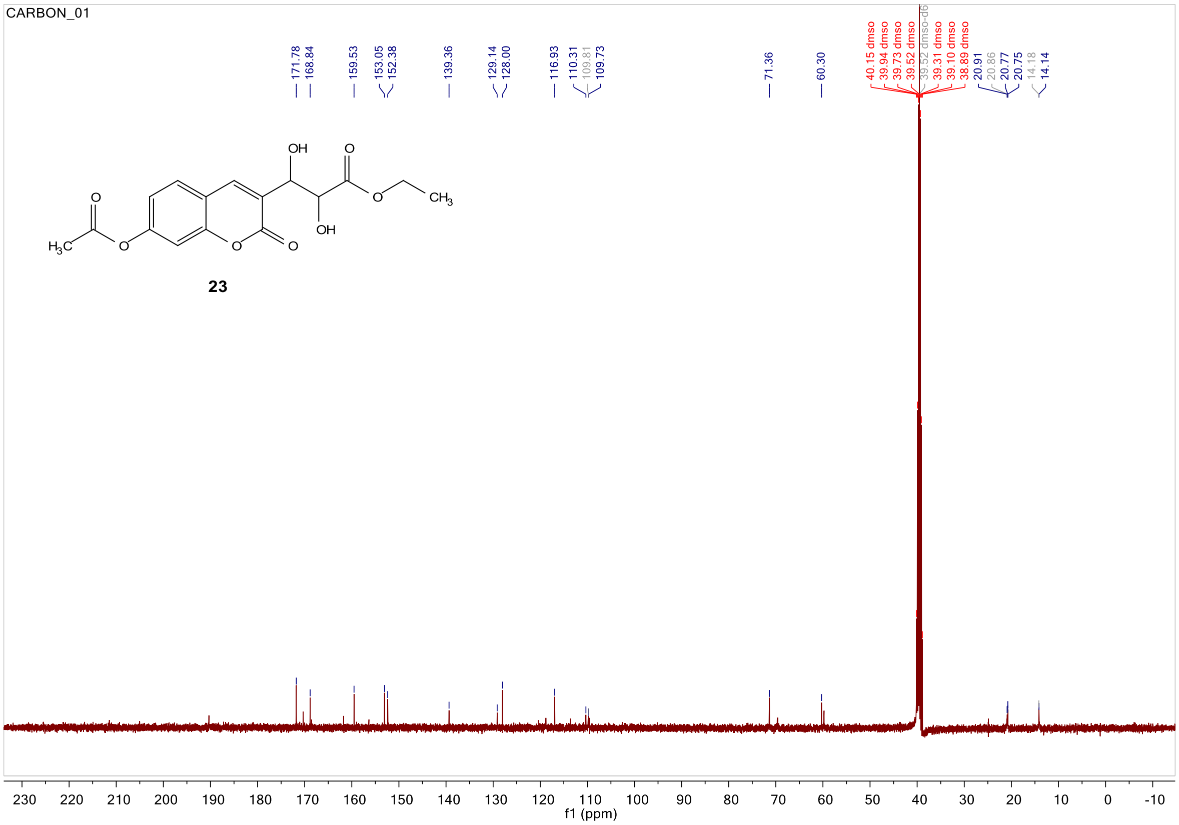

Supplementary Figure 26. ^{1}H and ^{13}C NMR spectra of **23.**

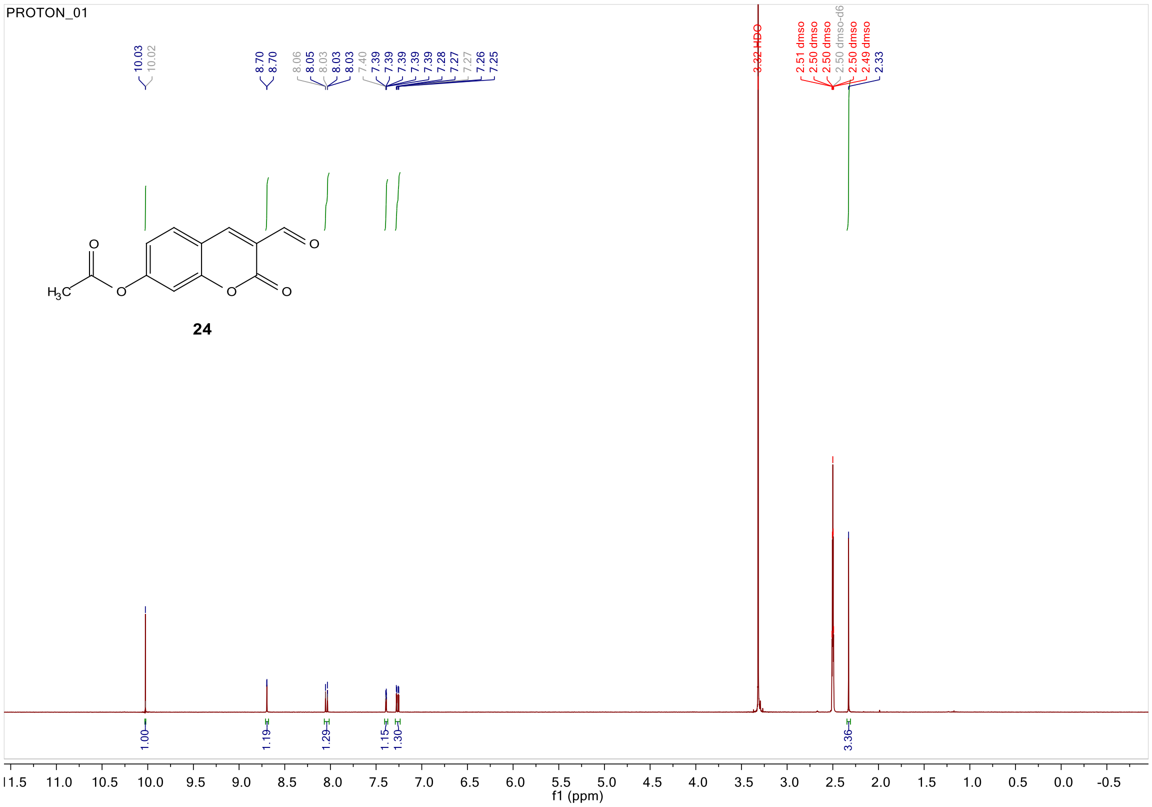

Supplementary Figure 27. ^{1}H NMR spectrum of **24.**

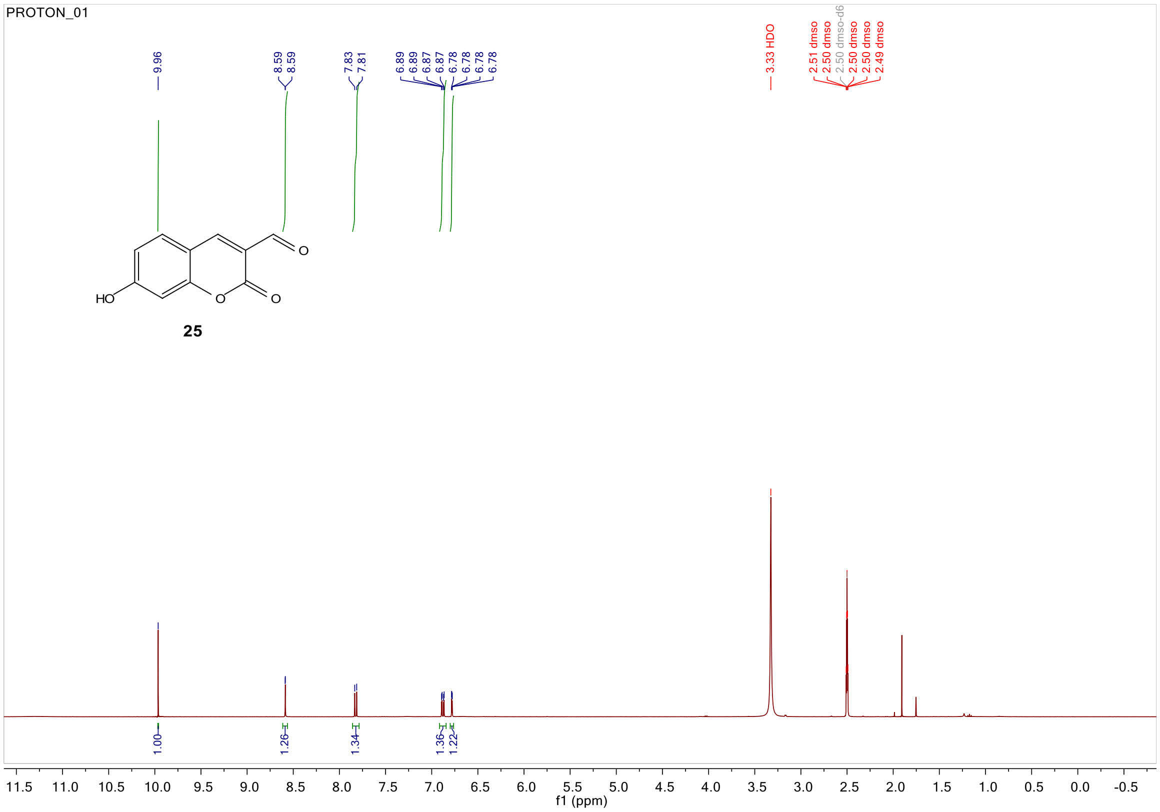

Supplementary Figure 28. ^{1}H NMR spectrum of **25**.
